# Host–Botrytis co-transcriptomics reveals finely tuned interactions with closely related legumes

**DOI:** 10.1093/g3journal/jkag125

**Published:** 2026-05-12

**Authors:** Anna Jo Muhich, Ritu Singh, Cloe Tom, Celine Caseys, Karishma Srinivas, Lucca Faieta, Brooke Grabbe, Daniel J Kliebenstein

**Affiliations:** Department of Plant Sciences, University of California Davis, Davis, CA 95616, United States; Department of Plant Sciences, University of California Davis, Davis, CA 95616, United States; Department of Plant Sciences, University of California Davis, Davis, CA 95616, United States; Department of Plant Sciences, University of California Davis, Davis, CA 95616, United States; Department of Plant Sciences, University of California Davis, Davis, CA 95616, United States; Department of Plant Sciences, University of California Davis, Davis, CA 95616, United States; Department of Plant Sciences, University of California Davis, Davis, CA 95616, United States; Department of Plant Sciences, University of California Davis, Davis, CA 95616, United States

**Keywords:** comparative co-transcriptomics, legumes, necrotrophic fungal pathogenesis, network biology, transcriptional plasticity, Fungal 2026

## Abstract

Generalist pathogens infect diverse plants, yet how these interactions differ across hosts is poorly understood. Here, we conduct a molecular analysis of a generalist pathogen interacting with closely related hosts. A co-transcriptomic framework is used to dissect host–pathogen interactions between the generalist necrotroph *Botrytis cinerea* and 2 closely related legume hosts, common bean (*Phaseolus vulgaris*) and cowpea (*Vigna unguiculata*). Using 72 diverse Botrytis isolates, we quantified lesion development alongside host and pathogen gene expression. Although lesion formation was driven primarily by pathogen genetic variation, transcriptomic responses in both host and pathogen exhibited significant host × isolate interactions. This indicated that extensive, fine-scale transcriptional plasticity created similar disease outcomes. Botrytis genes showing host-specific expression were enriched for cell wall–modifying enzymes and some specialized metabolic genes, indicating greater host responsiveness of these core virulence mechanisms than previously appreciated. Co-expression network analysis in both host and pathogen further showed that in both organisms, gene membership for individual networks is restructured in response to genetic diversity. Both legume species exhibited extensive isolate-dependent transcriptional reprogramming, with approximately two-thirds of expressed host genes responding to pathogen diversity. While conserved defense pathways such as jasmonate/ethylene signaling and phenylpropanoid metabolism were upregulated in both hosts, the specific genes in the networks differed markedly, highlighting lineage-specific rewiring of defense strategies. These results suggest that generalist pathogen success is underpinned by pervasive gene expression plasticity in both host and pathogen, allowing similar phenotypic outcomes to emerge from highly divergent molecular states.

## Introduction

Plant species rely on a diverse range of defensive strategies to defend against pathogens. These defenses are frequently multifunctional, allowing them to act against diverse pathogens. For example, chemical defenses like individual glucosinolates and monolignols, as well as physical defenses like callose deposition simultaneously provide resistance to multiple bacterial and fungal pathogens, both generalists and specialists, as well as insect herbivores (Liu et al. 2021; Ninkuu et al. 2022; Wang et al. 2021). Diverging plant species will often occupy different environmental niches that differ in the challenging pathogen populations, creating pressure on each species to separately optimize their defense portfolio. This can include changes in their specific defense strategies and/or signaling pathways controlling these defenses. Across distantly related species, SA (salicylic acid) and JA (jasmonic acid) signaling generally function as key response pathways to diverse biotic attackers. However, the production of the signals, the connectivity between the pathways, and the distinct downstream outputs often vary dramatically (Wang et al. 2015; Monte 2023). While this defense system variation occurs across distantly related species, the degree to which defense pathways vary between closely related plant species remains understudied.

How generalist pathogens successfully infect the broad range of hosts they encounter remains a compelling question. Key infection mechanisms include the production of digestive enzymes (Li et al. 2004), effector proteins that induce necrosis (Barrett and Heil 2012), enzymes that neutralize reactive oxygen species (López-Cruz et al. 2017), or phytotoxic metabolites (Colmenares et al. 2002). However, to infect many hosts, a generalist must either respond to the diversity of specialized defenses and shifts in host defense signaling or evade these defenses entirely. This suggests that successful generalists could rely on highly flexible or inducible counter-defense strategies that function across diverse host contexts. Somewhat counterintuitively, such flexibility implies generalists may deploy inducible specialized mechanisms that are expressed on specific hosts. There is some evidence for this in generalist pathogens, including specific defense metabolite efflux transporters (Stefanato et al. 2009). Recent studies have demonstrated extensive generalist pathogen transcriptional reprogramming when colonizing distinct hosts, including *Fusarium virguliforme* on maize and soybean (Baetsen-Young et al. 2020), *Sclerotinia sclerotium* on 6 eudicot families (Kusch et al. 2022), and *Botrytis cinerea* on 10 eudicot species (Singh et al. 2025). However, given the diversity of eudicot hosts, it remains unclear how finely tuned a generalist pathogen's virulence mechanisms are when comparing closely related host species infected by the same generalist pathogen.

To measure how host responses shift between closely related host plants and how a generalist pathogen compensates, we focused on the *B. cinerea*-legume pathosystem. *B. cinerea*, hereafter Botrytis, is a globally dispersed generalist fungal necrotroph with a host range spanning >1,400 land plant species, including vascular and nonvascular plants (Elad et al. 2016). Botrytis has extensive genome-wide sequence diversity that underlies complex quantitative disease outcomes across its many hosts (Rowe and Kliebenstein 2007; Fekete et al. 2012; Walker et al. 2015; Mercier et al. 2021; Caseys et al. 2021). Botrytis employs a wide array of virulence mechanisms, including phytotoxic metabolites, cell wall–degrading enzymes, and cell death–inducing proteins (Colmenares et al. 2002; Zhu et al. 2017), many of which exhibit substantial functional redundancy (Dalmais et al. 2011; Leisen et al. 2022). Further, alongside growing recognition that gene expression plasticity contributes to the success of generalist pathogens, increasing evidence indicates that Botrytis also undergoes extensive transcriptional reprogramming in response to different hosts. When Botrytis gene expression was compared on wild-type *Arabidopsis thaliana* vs SA/JA signaling mutants, 45% to 74% of the detectable Botrytis transcriptome was differentially expressed across host genotypes (Zhang et al. 2019). Much of its expression variation was attributable to differences in Botrytis isolate and was largely controlled in trans, suggesting coordinated regulatory programs rather than gene-by-gene responses (Krishnan et al. 2023). In some fungi, including Botrytis, it is possible to simultaneously measure host and pathogen transcriptomes during infection using a co-transcriptomic approach. This framework has been previously applied in Arabidopsis, revealing correlated host–pathogen gene co-expression networks and identifying novel mechanisms underlying the Arabidopsis–Botrytis interaction (Zhang et al. 2019). Co-transcriptomics in Botrytis therefore provides a powerful platform for dissecting the reciprocal strategies of host and pathogen during infection and can be extended to other agronomically important hosts.

Among Botrytis's many hosts are the Fabaceae or legume family in the rosids clade. Legumes are a widely grown crop and are of growing importance for sustainable agriculture (Voisin et al. 2014). Common bean (*Phaseolus vulgaris*) and cowpea (*Vigna unguiculata*) are 2 closely related agronomically important legumes, diverging ∼10 Mya (Moghaddam et al. 2021). As known Botrytis hosts, they provide a model for comparing host response specificity of closely related plants when challenged with the same pathogen. Legumes represent the third largest plant family and display a massive diversity of potentially toxic specialized metabolites, including alkaloids, nonprotein amino acids, cyanogens, peptides, phenolics, polyketides, and terpenoids (Wink 2013). In common bean and cowpea, both species likely use flavonoids and other phenolic compounds for defense, but comprehensive metabolomic studies for these species are minimal (Perez De Souza et al. 2019). Due to the large diversity of legume bioactive metabolites, it is possible that their respective defense strategies diverge in the specialized metabolic pathways they utilize against the same pathogen.

In this study, we used the legume–Botrytis pathosystem to map the host-specific aspects shaping the interaction when comparing closely related hosts. This involved first measuring Botrytis virulence on different genotypes of legumes. Lesion sizes were quantified using 72 diverse *B. cinerea* isolates on 4 genotypes each of common bean and cowpea. For a molecular investigation, the co-transcriptomes of all 72 diverse Botrytis isolates and 1 genotype each of common bean and cowpea were measured during infection. This provided a map of how gene expression patterns in both the host and pathogen contribute to virulence, as well as the identification of Botrytis genes regulated in a host-specific manner across diverse isolates.

## Results

### Lesion size and phenotypic variation

**Fig. 1.**
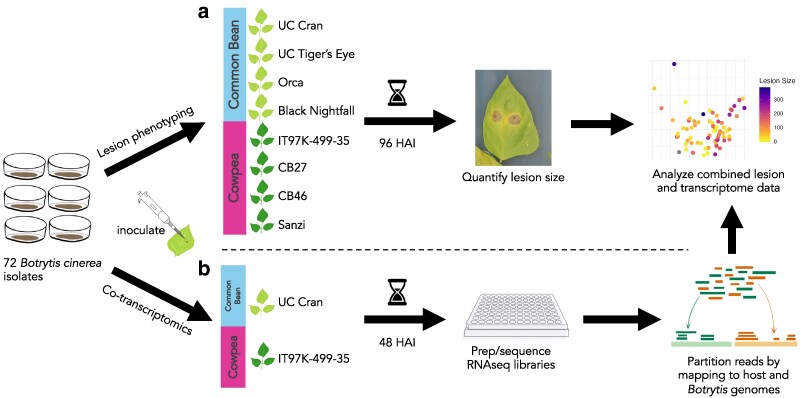
Schematic of experimental design. Lesion phenotyping was conducted across 2 independent experiments, each with 3 biological replicates, and transcriptomics was conducted as one experiment with 3 biological replicates. HAI, hours after inoculation.

We measured Botrytis lesion formation on cowpea and common bean to test how the difference between closely related hosts may influence host–Botrytis interactions. A total of 72 diverse Botrytis isolates were individually inoculated on leaves from 4 representative genotypes each of common bean and cowpea (Fig. 1). These isolates are known to drive disease outcomes on alternative hosts (Caseys et al. 2021; Singh et al. 2025) and are used to distinguish stable host responses vs those conditional on pathogen variation. Using a long-established assay for detached leaf assays (Denby et al. 2004; Caseys et al. 2021), the developing lesions were imaged at 24, 48, 72, and 96 hours after inoculation (HAI), and digitally quantified at 72 and 96 HAI when lesions became visible. Lesions on common bean and cowpea grew quickly from 72 to 96 HAI, but the rank order of the Botrytis isolate virulence between these timepoints was largely retained (Supplementary Fig. 1). To capture variation in lesion progression across isolates, lesion areas at 96 HAI were used for further analysis.

**Fig. 2.**
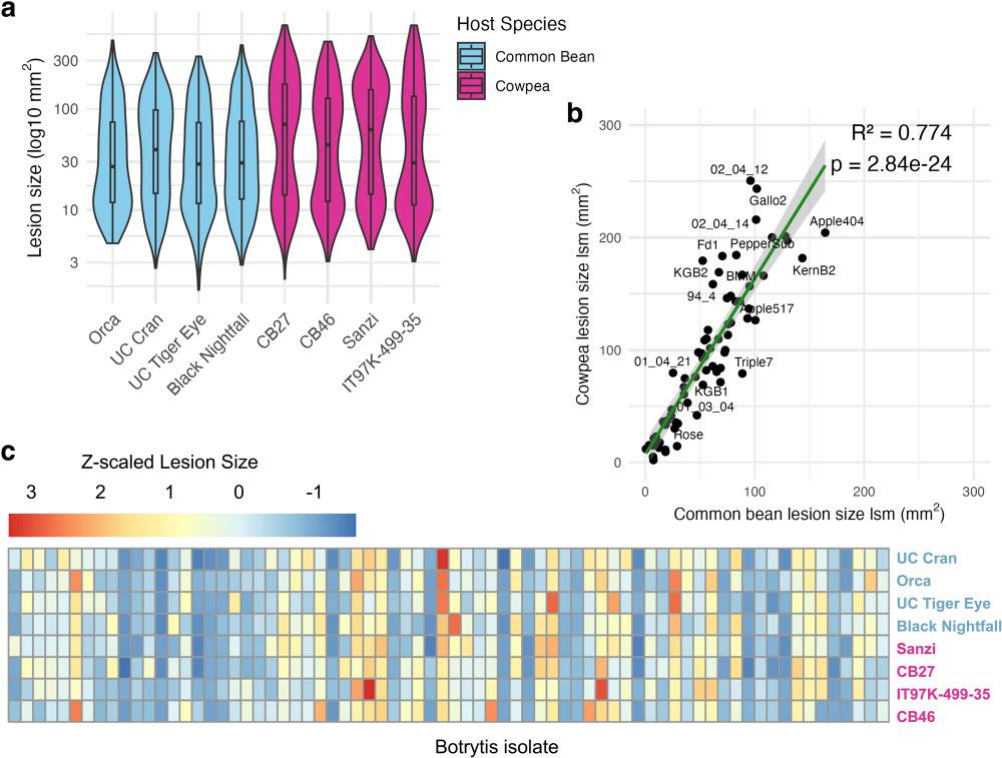
Lesion sizes of 72 *Botrytis* isolates on 2 legume host species at 96 HAI. a) Violin plot shows the overall distribution of lesion sizes collected for each host genotype. b) Correlation of host lesion size for each isolate. Species lesion size was calculated as the least-square mean (lsm) across the host genotypes. Outlying isolates are labeled with the isolate name. c) Lesion size heatmap showing z-scaled lesion sizes for each individual host genotype and Botrytis isolate. Common bean genotypes are colored in blue, and cowpea genotypes are colored in magenta.

While cowpea showed slightly higher lesion size overall at 96 HAI compared to common bean, the genotypes within each host species showed a similar distribution of lesion sizes across the diverse Botrytis isolates (Fig. 2a). This was reflected when conducting a linear model combining the lesion data across both host species. In this model, the terms host genotype, host species, and Botrytis isolate all significantly contribute to lesion size, where isolate was the strongest contributor (Supplementary Table 1). In agreement with the strong isolate effect in the model, the lesion formation of the isolates was linearly correlated across the 2 species (Fig. 2b). Interaction terms for host genotype × Botrytis isolate and host species × Botrytis isolate were not significant, indicating that we detected minimal variation of individual isolate's lesion size across different hosts (Supplementary Table 1, Fig. 2c).

To quantify how the Botrytis isolates' lesion formation differs across host genotype variation for both host species, we calculated average species lesion residuals for each isolate (Supplementary Fig. 2). This was calculated for each isolate by subtracting the average lesion size for the isolate on a host species from its mean on each specific host genotype. These host genotype-level residual absolute values were then summed to generate one host species-level residual value for each host species and isolate combination. Therefore, an isolate with a residual value near 0 has low within-species specificity, while an isolate with a residual value far from 0 has high within-species specificity. This showed that several isolates are more sensitive to within-host species genetic variation, but the correlation across host species was only moderate (*R*^2^ = 0.458), indicating that for many isolates, host species is not the most important variable. Together, these results suggest that host–Botrytis interactions in these 2 closely related legumes are largely driven first by variation in the pathogen, and that secondly, variation within the respective host species is frequently a more important factor than across species.

### Botrytis transcriptome differs across 2 legume host species

Since the observed disease outcomes between closely related hosts were similar to each other, we wanted to test if the underlying transcriptional machinery governing this outcome is also similar between hosts. To test this, we next proceeded to measure the transcriptomic responses of the 72 Botrytis isolates against one selected genotype from each cowpea and common bean. The host genotypes were selected based on several factors, including range of infection phenotypes across the Botrytis isolates, available reference genomes, and ease of healthy plant growth. The co-transcriptome was obtained by extracting total mRNA from the developing lesion at 48 HAI and sequencing both the host and pathogen transcriptomes from the same samples and libraries (Fig. 1). On average, samples yielded 11 million total reads, with 78% mapping to the host genome and 10% mapping to the Botrytis genome (Supplementary Table 2). Due to the lack of long-read references for all 72 Botrytis isolates, all Botrytis reads were mapped to the B05.10 reference genome (van Kan et al. 2017). The isolate variation missed with this approach is expected to be minimal as previous analysis has shown a low level of gene presence/absence variation in this collection (Caseys et al. 2021; Singh et al. 2025). The following section focuses solely on analyzing the pathogen's transcriptome.

**Fig. 3.**
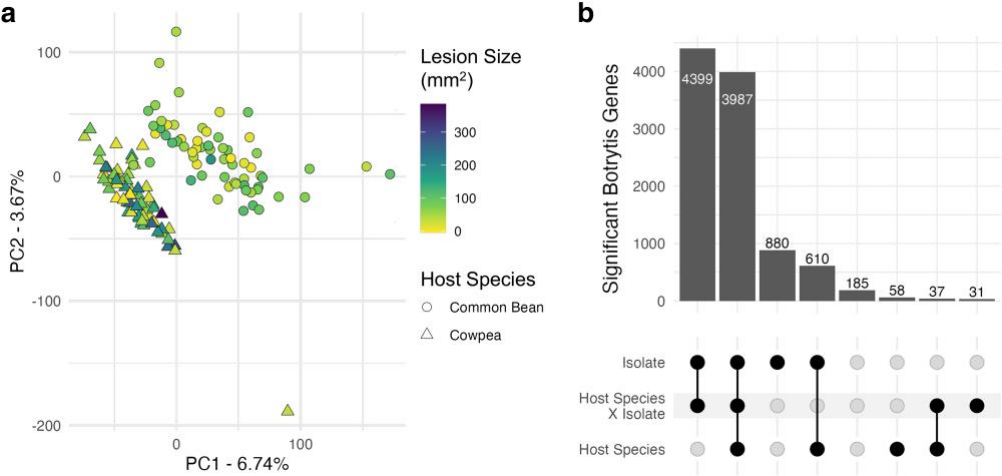
Overall Botrytis transcriptome variability during infection of 2 legume host species. a) PCA of overall Botrytis transcriptome at 48 HAI. Shape of points shows the species the infecting Botrytis isolate was collected from, where circles are common bean and triangles are cowpea. Points are colored by lesion size of that Botrytis isolate on that host species at 96 HAI. b) Upset plot summary of ANOVA for each Botrytis gene expression on legume hosts at 48 HAI. Botrytis genes were separately modeled with the formula: gene expression ∼ host species + isolate + (host species * isolate). Genes are included in each category if the FDR < 0.05 for that model term. Genes with little to no expression are not diagrammed and include 1,887 Botrytis genes.

Principal component analysis (PCA) of Botrytis gene expression means showed that the overall Botrytis transcriptome had a difference in response to the 2 host legumes (Fig. 3a). Comparing these transcriptome vectors to lesion formation in the 2 hosts showed that the main axis of Botrytis isolate transcriptome variation (PC1) does not significantly associate with lesion size (*P* = 0.22), but the second axis (PC2) does significantly associate to lesion size (*P* = 0.003). However, PC2 is only 3.67% of the overall variance in the Botrytis transcriptomic dataset, suggesting that Botrytis gene expression is not the only contributing factor to disease outcomes. PC2 also significantly associates with the total Botrytis transcript abundance (total number of mapped Botrytis reads) for the sample (*P* < 0.001) (Supplementary Fig. 3). This analysis shows that while Botrytis transcriptomic variation between hosts was evident, only a small proportion of the variation captured by the leading principal components was significantly associated with lesion size and overall transcript abundance. Additional variation represented in the higher-order components may also contribute, alongside other biological or environmental factors.

Moving beyond the whole transcriptome level to the individual transcript level, 10,187 out of 12,111 total genes in the Botrytis genome could be quantified after quality control and low readcount filtering as defined in the methods. The vast majority of Botrytis genes (83%) were differentially expressed during infection, either between the host species, amongst the Botrytis isolates, or across the interaction. Assessing by the number of genes associated with each term, Botrytis gene expression variation across the dataset was first influenced by the Botrytis isolate, secondarily by host species × Botrytis isolate interaction, then thirdly by host species (Fig. 3b). This result contrasts with the lesion modeling, which showed little to no host × isolate interactions in lesion formation. With a large portion of genes having a host × isolate effect on their expression, this suggests a large amount of variation of underlying host × isolate effects on gene expression was masked beneath the lesion phenotype.

To determine the contribution of individual Botrytis gene expression on the lesion phenotype, a linear model was built for each gene's expression with respect to the host using lesion size ∼ host + expression + host × expression (Supplementary Fig. 4a). All Botrytis gene expressions showed low correlation with gene expression (*R*^2^ < 0.3), most of which were not significant (*P* > 0.05). Four Botrytis genes (Bcin03g05160, Bcin08g05330, Bcin12g04440, and Bcin14g00040) had significant weak correlations in interactions with the host, but these lack strong annotation information or clear roles in virulence. Therefore, expression levels of individual Botrytis genes have little to no detectable effect on the lesion phenotype.

### Differential expression of Botrytis genes across hosts highlights host-specific virulence mechanisms

**Fig. 4.**
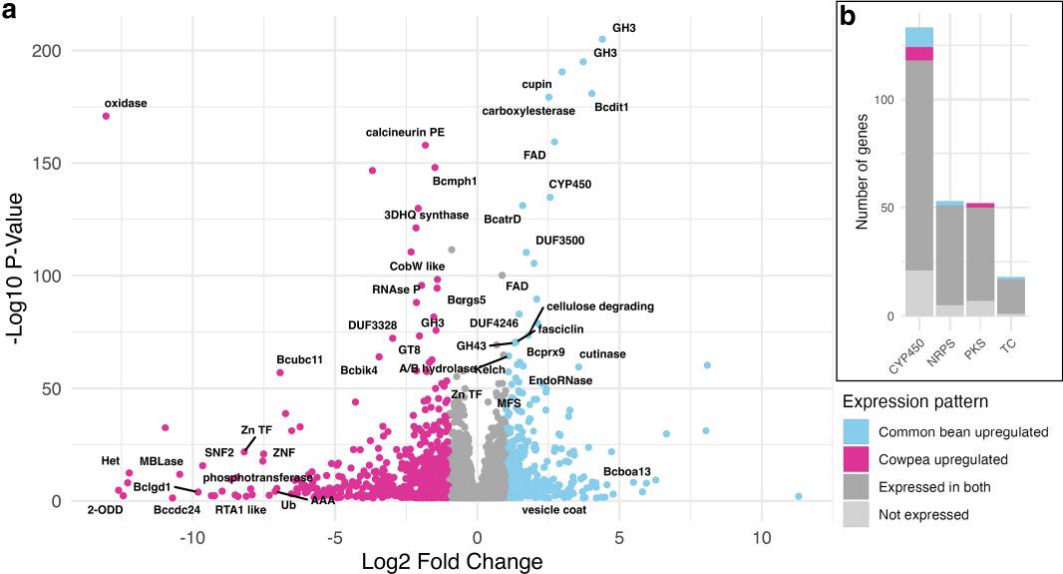
Host-specific expression of Botrytis genes. a) Volcano plot of Botrytis genes plotted by the gene's log2 fold change value when infecting common bean vs cowpea. Annotated genes with -Log10 *P*-value > 50, log2FC > 4, or log2FC < -7 are labeled on the plot. b) Count of genes in key secondary metabolic gene classes for Botrytis, colored by their host-specific expression patterns. CYP450, cytochrome P450; NRPS, nonribosomal peptide synthase; PKS, polyketide synthase; TC, terpene cyclase.

We hypothesized that the Botrytis genes showing a large difference in expression between common bean and cowpea could provide insight into how Botrytis's virulence strategy might change across these closely related hosts. Of the 4,692 Botrytis genes with a significant host effect, only 58 Botrytis genes had solely a host effect with no isolate variation. GO enrichment of these 58 genes against the whole Botrytis genome showed enrichment in protein binding and endoribonuclease activity (Supplementary Table 3). To look more broadly at host-specific Botrytis genes, we focused on all 4,692 genes with a host effect regardless of whether they also had an isolate effect, as this offered a more inclusive assessment of the differences in response to the hosts. Filtering the 4,692 total Botrytis genes with a host species effect for those with at least a 2-fold difference between the 2 hosts yielded 1,078 differentially expressed Botrytis genes (Supplementary Table 4). This included 656 Botrytis genes significantly upregulated during infection of cowpea in comparison to common bean, and 422 Botrytis genes significantly upregulated during infection of common bean in comparison to cowpea (Fig. 4a). While GO enrichment of each host-specific group showed similar results (Supplementary Table 5), many of the most differentially expressed genes are in gene families known to aid in cell wall penetration and host surface modification. Several identified genes encode functions like glycosyl hydrolases, glycosyl transferases, cellulose degrading, cutinases, etc. This suggests that Botrytis may be responding to common bean and cowpea differently by altering cell wall-degrading gene expression. In addition to cell wall metabolism, a number of genes are involved in unknown specialized metabolite pathways, including polyketide synthases and cytochrome P450s.

To measure the relative host specificity of Botrytis specialized metabolic gene expression, we used a previously described annotation of known specialized metabolic enzymes in Botrytis (Suárez et al. 2024). This allowed for classification of enzymes into 4 major categories: cytochrome P450s (CYP450), nonribosomal peptide synthases (NRPS), polyketide synthase (PKS), and terpene cyclases (TC), including both diterpene and sesquiterpene cyclases. All genes in each specialized metabolic class were totaled within their observed regulatory pattern groups on the hosts (up on common bean, up on cowpea, up on both hosts, or up on neither host). While most of these specialized metabolic genes were expressed when infecting both hosts, a small number of genes in each class showed host specificity (Fig. 4b). This indicates Botrytis displays small adjustments in specialized metabolic pathways even when infecting closely related hosts. Each regulatory pattern gene group (upregulated in common bean only, upregulated in cowpea only, upregulated in both, or upregulated in neither) was then tested for enrichment of these 4 specialized metabolic classes against the ratios of specialized metabolic genes present in the whole genome using the hypergeometric distribution. No single specialized metabolite class was significantly enriched during infection of either host, indicating that Botrytis uses a blend of specialized metabolites depending on the host overall.

### Key Botrytis phytotoxin pathways express similarly on legume hosts across diverse isolates

In addition to differences between the hosts, genetic variation between the 72 Botrytis isolates was a major driver of lesion and Botrytis transcriptome variation (Fig. 3b). To better understand which Botrytis virulence mechanisms may be affected by isolate variation, yet likely function equally across the hosts, we generated a list of the 880 Botrytis genes with a significant isolate effect and no significant host-associated variation. This list represents genes that vary in expression across isolates but are expressed similarly when infecting either host species. To obtain a clearer mechanistic signal from this large list of genes, Botrytis gene co-expression networks (GCNs) were generated separately using each of the 2 host datasets using all expressed genes. These GCNs were filtered for those where at least 25% of genes in the GCN are from the list of 880 Botrytis isolate-specific genes to identify common Botrytis GCNs expressed similarly across hosts, but altered by the pathogen's genetic variation. These identified 18 isolate-specific Botrytis GCNs, including 10 found when infecting common bean and 8 when infecting cowpea (Supplementary Table 6). Among these networks was one including BcVEL1, a gene from the VELVET regulatory complex known to be genetically variable and influence virulence across hosts (Schumacher et al. 2012).

**Fig. 5.**
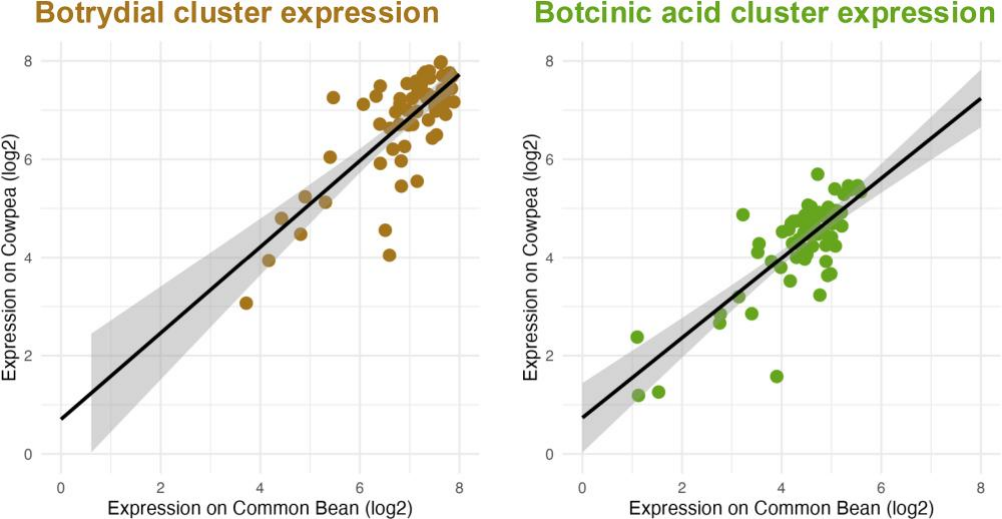
Expression of Botrytis phytotoxin biosynthetic pathways across host species. Average z-scaled expression (log2) was calculated for known genes in each pathway for each Botrytis isolate. Host-specific expression of each isolate was plotted. Botrydial pathway consisted of a 5-gene cluster (Bcin12g06370-Bcin12g06410), while botcinic acid pathway consisted of a 16-gene cluster (Bcin01g00010-Bcin01g00160).

In this list of similarly expressed GCNs across hosts, there were also Botrytis GCNs that included the well-described biosynthetic pathways for key phytotoxins, botrydial and botcinic acid. These biosynthetic pathways exist as 2 gene clusters, each co-localized on separate chromosomes (Supplementary Table 7). The expression of these phytotoxin clusters exhibits a quantitative and qualitative variation across the isolates, where most isolates express the phytotoxins on both hosts, and several isolates have very low or no expression of one or the other pathway on either host (Fig. 5). This suggests Botrytis expresses botrydial and botcinic acid similarly across both closely related hosts, and these virulence mechanisms may be an example of a generalized defense. Still, individual isolates varied widely in their expression of these pathways, and the variation in the 2 phytotoxins was independent, such that there are a few isolates that have lost or dramatically decreased the expression of one cluster or the other on a specific host. However, the isolates that have low expression of these phytotoxin pathways overall do not have apparently reduced virulence (Supplementary Table 8) and are likely relying on other mechanisms to induce host toxicity, consistent with previously reported functional redundancy of these phytotoxins (Dalmais et al. 2011; Leisen et al. 2022).

### Botrytis gene co-expression networks vary across host and isolate genetic diversity

Most Botrytis genes' expression has a significant host × isolate interaction, meaning their expression varies across the hosts depending on which isolate is infecting the hosts. The observation that host × isolate interactions had minimal influence on lesions but large effects on the transcriptome suggested 2 possible models. First, each host induces different Botrytis networks that are variable across isolates and sum to create similar lesion variation. In the second model, the host effects reshape similar networks across the isolates, leading to similar lesions. To test these models, we assessed if the host × isolate Botrytis GCNs found on cowpea and common bean were different (model 1) or contain subsets of the same genes (model 2).

**Fig. 6.**
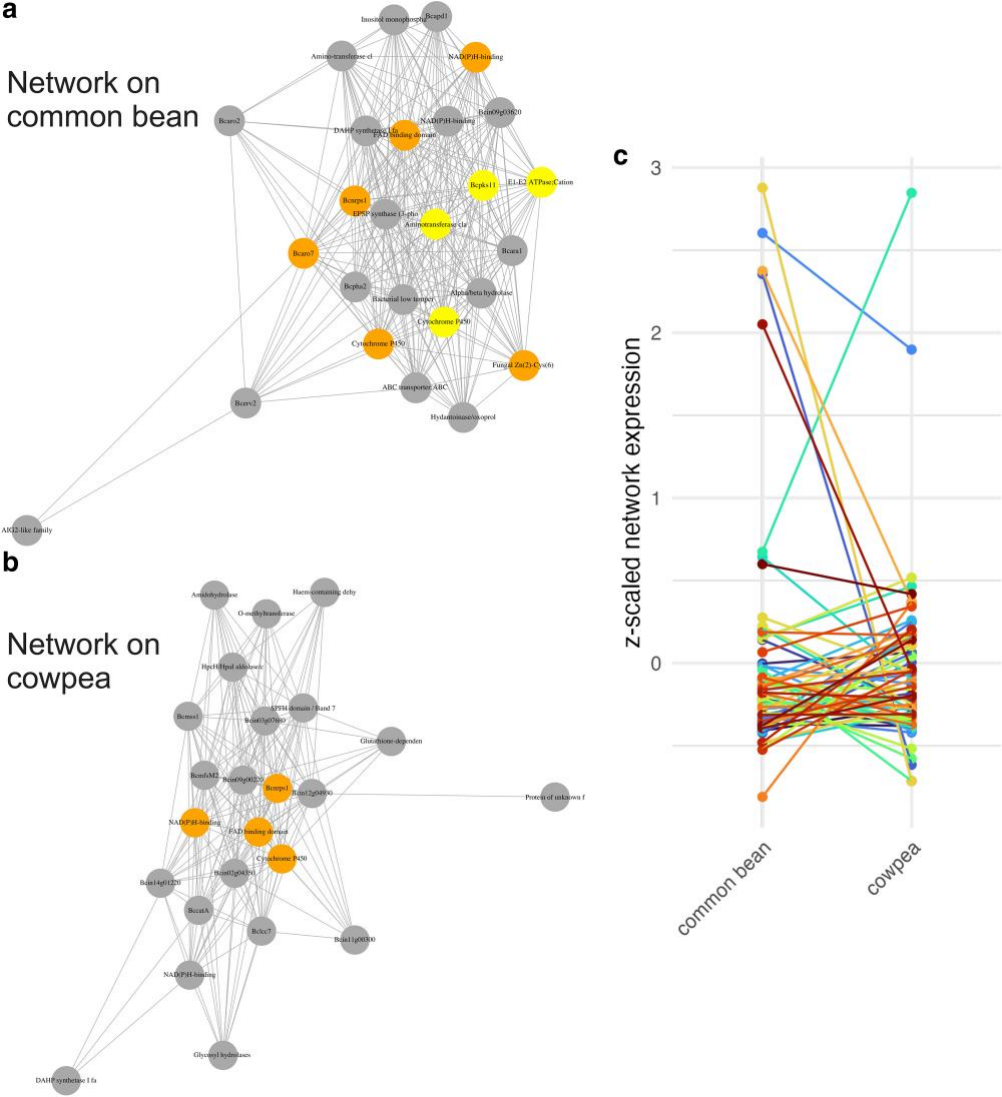
Selected Botrytis co-expression networks containing several genes with significant host × isolate interaction. Overlapping Botrytis gene co-expression networks were detected when infecting both a) common bean and b) cowpea with slight differences. Biosynthetic gene clusters detected within each network are highlighted in color, where the NRPS cluster on chromosome 12 is in orange, and the PKS cluster on chromosome 14 is in yellow. c) Z-scaled expression of these interaction networks on different hosts. Colored points and connecting lines represent different Botrytis isolates.

First, the full set of Botrytis GCNs was filtered to identify networks that contained at least 5 of the top 100 host × isolate interaction-significant Botrytis genes based on *P*-value of the interaction term. This approach found 4 Botrytis GCNs when infecting common bean and 5 when infecting cowpea. Because the Botrytis GCNs represent infection of 2 different host datasets, GCNs that contain similar sets of genes can be identified on either host. Among this set of 9 Botrytis GCNs were 2 pairs of overlapping GCNs, each containing some of the same gene members across the hosts. The first overlapping Botrytis GCN found on both host species identifies a co-expressed and previously undescribed gene cluster on chromosome 13 that may be responsible for breakdown and potentially detoxification of host compounds (Supplementary Fig. 5). The second overlapping Botrytis GCN centers around a nonribosomal peptide synthase (NRPS) gene cluster on chromosome 12 (Fig. 6a & b). Comparing z-scaled expression values of these networks for each host–isolate combination shows large differences in network expression across hosts for several isolates (Fig. 6c). In addition to differences in expression on the hosts, gene memberships in the networks can shift. While these 2 GCNs have some identical gene members shared across both hosts, there are key differences in overall network structure when infecting common bean vs cowpea. Interestingly, when infecting common bean, the NRPS cluster co-expresses with several shikimate pathway genes (EPSP synthase, BcPHA2, BcARO2, and BcARO7) as well as an additional polyketide synthase (PKS) gene cluster from chromosome 14 (Fig. 6a). Many fungi have been shown to have hybrid PKS–NRPS enzymes that catalyze steps in flavonoid biosynthesis, and can do so directly from p-coumaric acid (Zhang et al. 2022). However, when infecting cowpea, the Botrytis PKS gene cluster GCN lacks co-expression with these shikimate-related enzymes. Instead, the network is modified such that the NRPS cluster co-expresses most closely with several tailoring enzymes that could modify an NRPS-based metabolite, including an O-methyltransferase, amidohydrolase, laccases (CLCC2 & CLCC7), and dehydratases (Fig. 6b). This may describe a system whereby the core PKS-NRPS module has different potential tailoring/modification genes depending on the host and the isolate. These modified NRPS networks provide an example of a Botrytis response system potentially encoding unknown branching metabolic pathways that can be observed across genetically diverse interactions, but differentially fine-tuned depending on both the host genotype and the specific Botrytis isolate. While further study is needed to confirm the biosynthetic potential of these gene clusters, these examples and the lack of any completely overlapping Botrytis GCNs between hosts support the second model proposed above: closely related hosts reshape similar Botrytis networks across the isolates, but these changes lead to similar lesion variation. Future work can show if these network restructuring patterns are unique examples or part of a broader trend in host specificity.

Although expression levels of individual Botrytis genes did not strongly contribute to lesion size (Supplementary Fig. 4a), we wanted to determine if these NRPS networks or others have higher expression levels in more virulent isolates. The average network expression was calculated for all networks on the top 10 and bottom 10 isolates in lesion rank order (Supplementary Fig. 4b). This showed that Botrytis isolates that made smaller lesions had approximately equivalent average network expression of each network to isolates that made larger lesions. Therefore, none of the Botrytis GCNs strongly explain the differences observed between the most extreme lesions.

### Host species transcriptomes response to Botrytis infection is shaped by Botrytis genetic diversity

**Fig. 7.**
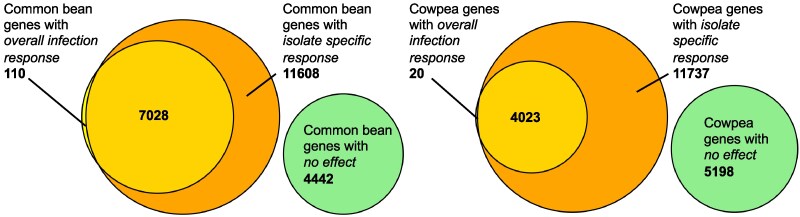
Summary of ANOVA for each legume host gene expression at 48 HAI with Botrytis. Host genes were separately modeled with the formula gene expression ∼ infected + infected/isolate. Genes are included in each category if FDR < 0.05 for that model term. Genes with little to no expression are not diagrammed and include 11,273 genes for common bean and 14,993 genes for cowpea.

The co-transcriptome approach allows us to simultaneously measure the hosts' gene expression in these exact same samples and compare and contrast the host's response with the pathogen's transcriptome. For each host species, there was a single genotype used, and time-matched uninfected samples were included as controls. Each host gene's expression was modeled with the formula: *gene expression ∼ infected + infected/isolate*. In contrast to the Botrytis model, this had to be run independently on the 2 hosts as the genes are different in the 2 species. The proportion of genes that responded significantly to infection overall was considerably higher in common bean (26%) than in cowpea (12%). However, in both hosts, a large proportion of host genes were differentially expressed in response to the diverse isolates, 42% (11,608 genes out of 27,433 total) for common bean and 37% (11,737 genes out of 31,948 total) for cowpea (Fig. 7). This is in agreement with variation in Botrytis isolate being a key determinant of lesion formation in both hosts. A key difference between the 2 host models is that a higher proportion of genes respond to infection overall in common bean than in cowpea. This suggests that although overall variance in host gene expression across isolates is similar between the 2 species, cowpea exhibits a weaker average transcriptional response to Botrytis than common bean. This may partially explain the slightly larger average lesion size for cowpea (Fig. 2a).

To assess the overall behavior of the 2 host transcriptomes, we initially conducted PCAs that showed the PC1 in common bean explained 39% of its transcriptomic variation, while the PC1 in cowpea explained 42% of its transcriptomic variation (Supplementary Fig. 6). In both hosts, PC1 associates with the estimated Botrytis biomass (as measured by total reads mapped to the pathogen; Botrytis transcript abundance) (Supplementary Fig. 6a). Interestingly, PC1 in either host does not associate with lesion size at 96 HAI (Supplementary Fig. 6b). Because host gene expression is not strongly associated with disease incidence but is instead more responsive to the abundance of Botrytis transcripts this suggests that host response during early infection is influenced primarily by variation in the Botrytis isolate. This agrees with previous observations that lesion formation and Botrytis biomass are only mildly correlated (Corwin, Subedy et al. 2016).

### Host orthology analysis reveals several conserved patterns in response to Botrytis

To enable a direct comparison of host responses between the 2 host species, we conducted a genome-wide orthology analysis to procure a list of single-copy orthologs between common bean and cowpea. This identified 18,913 genes that are single-copy orthologs between common bean and cowpea. These are genes that have a single gene match across the legume species and allow us to directly compare the transcriptome response to Botrytis between both host species. A majority (∼70%) of the expressed host genes in the study comprise the list of single-copy orthologs, a representative subset of both the cowpea and common bean genome (Supplementary Fig. 7). In either host, nonorthologs have a similar proportion and magnitude of response to infection compared with single-copy orthologs (Supplementary Fig. 8). While nonorthologs are biologically relevant to host defense, we focused largely on single-copy orthologs to compare host responses directly.

**Fig. 8.**
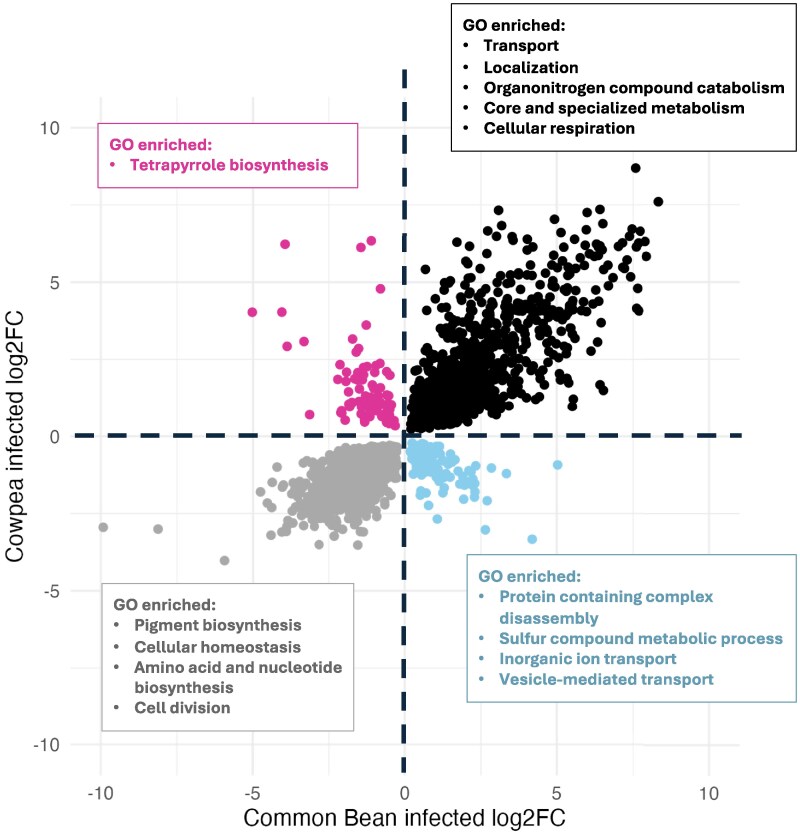
Orthology reveals shared and host-specific responses to Botrytis. Comparative log2FC of single-copy orthologs between cowpea and common bean that have a significant response to infection in both host species. Log2FC is calculated as the difference in expression from control to infected. Genes are colored by their expression pattern, where magenta is upregulated in cowpea and downregulated in common bean, blue is upregulated in common bean and downregulated in cowpea, black is upregulated in both, and grey is downregulated in both. Summary of significantly enriched GO terms (*P* < 0.05) is shown for each quadrant. Only genes significantly altered by infection are shown (FDR < 0.05).

Of the 18,913 single-copy orthologs, 2,923 were expressed in common bean, but not cowpea, 2,509 were expressed in cowpea, but not common bean, and 5,294 were not expressed in either host. The remaining 8,187 single-copy orthologs expressed in both hosts were then filtered to the 2,541 that had a significant response to Botrytis infection in both hosts. Using this list of orthologs, we directly compared the 2 host species' transcriptomic responses to Botrytis infection by calculating the mean expression of each gene in the mock sample and across all the Botrytis isolates. This was used to calculate the log2 fold change (log2FC) of each transcript in response to infection within each host species. Plotting the log2FC values of each single-copy ortholog across the 2 host species allowed for splitting the genes into 4 quadrants: upregulated in both hosts, downregulated in both hosts, upregulated in common bean but downregulated in cowpea, and downregulated in common bean but upregulated in cowpea (Fig. 8, Supplementary Table 9). This showed that while most host orthologs show similar responses to infection, there is a set of genes with opposite responses to infection in the 2 host species.

GO analysis on the 1,235 genes upregulated by Botrytis infection in both host species identified 86 significantly enriched GO terms. The most significant terms were dominated by those related to localization and transport, but many significant terms also included cellular respiration and core cellular metabolic processes, including terms for mono- and dicarboxylic acids, pyruvate, nucleotides, etc. (Supplementary Table 10). Additionally, several pathways producing specialized metabolite precursors were upregulated in both hosts, including malonate and isoprenoid biosynthesis. These pathways potentially feed into the downstream production of flavonoids and terpenes. Defense signaling pathways were also upregulated in both hosts, including jasmonic acid pathway genes (JAZs, LOX3, OPR3) and ethylene pathway genes (ACC synthase, ERF1).

Testing the 1,088 single-copy orthologs downregulated in both hosts during infection identified 39 GO terms. Overall, these terms were more representative of processes associated with photosynthesis and growth, including pigment, amino acid, and nucleotide biosynthesis, cell division, and cellular homeostasis. These processes are also downregulated in other diverse host plants when infected by Botrytis (Windram et al. 2012; Zhang et al. 2017, 2019; De Cremer et al. 2013).

There were 218 single-copy orthologs differentially responsive in the 2 species (Fig. 8). Relatively few genes were downregulated in common bean but upregulated in cowpea, with only 2 enriched GO terms both relating to tetrapyrrole biosynthesis. For those genes upregulated in common bean but downregulated in cowpea, these were enriched for 5 GO terms, including complex disassembly, sulfur compound metabolism, and transport.

### Co-expression analysis reveals differences in upregulated host responses to Botrytis

To provide more information on the processes represented in the single-copy orthologs in the genomic contexts of both hosts, we used the host GCNs. The host GCNs were determined separately for each host species so that networks could be built with both orthologous and nonorthologous genes within each host. The networks were filtered to those that had at least 10 members, and had at least 50% of their members as a host single-copy ortholog significantly altered by infection in both hosts (same genes as Fig. 8). This resulted in 37 infection-responsive host networks, 20 for common bean and 17 for cowpea. Approximately half of these networks contained orthologs that were downregulated in both hosts, mainly annotated as photosynthetic genes (Supplementary Table 11).

**Fig. 9.**
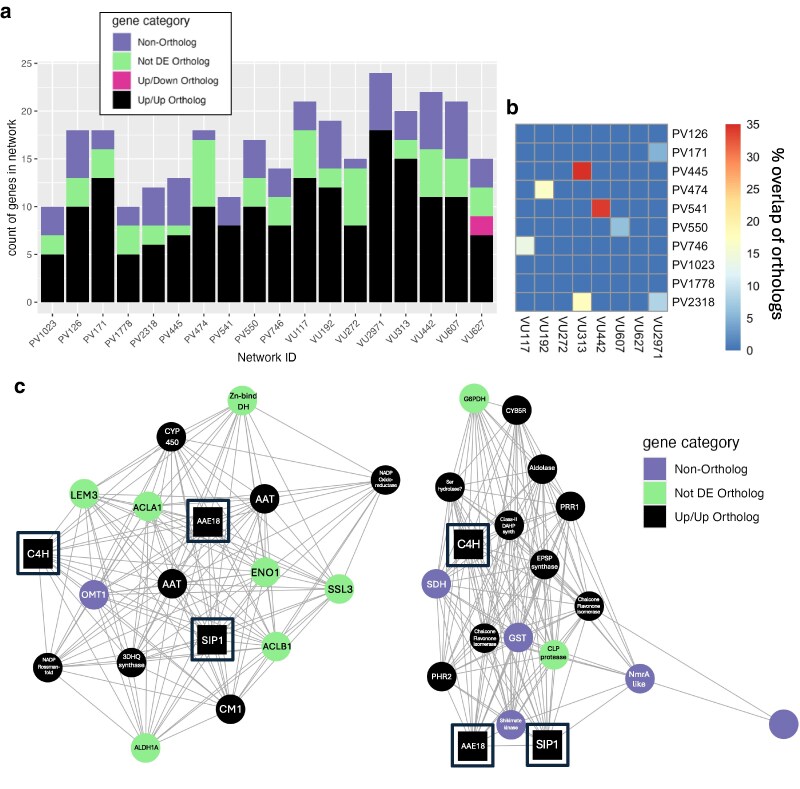
Upregulated host networks share similarities in overall function, but high variability in gene membership across host species. a) Total genes present in each upregulated host network, colored by orthology and regulatory category. Up/up ortholog = upregulated in both hosts during infection, up/down ortholog = upregulated in one host and downregulated in the other during infection, not DE ortholog = not differentially expressed during infection, and Nonortholog = a gene without single-copy orthology between the hosts. b) Heatmap showing network homology via the percent of overlap among single-copy orthologs across networks. c) Upregulated phenylpropanoid biosynthetic GCNs from common bean (PV474) and cowpea (VU192) with homology across hosts. Squares represent single-copy orthologs with overlap across the networks.

To look more closely at processes upregulated in response to Botrytis in both hosts, we focused on the upregulated networks, which included 10 for common bean and 8 for cowpea. These networks contained a blend of single-copy orthologs and other genes specific to each host (Fig. 9a). Since the majority of the network members were single-copy orthologs upregulated in response to Botrytis in both hosts, we hypothesized that the genes are similarly regulated in either host. Therefore, we expected that corresponding networks across hosts would share a high fraction of genes. However, we found a relatively low degree of similarity in gene content between the upregulated host networks (Fig. 9b). The 5 most similar networks (ranging from 14% to 35% match) all resembled various transport/signaling pathways, with the exception of 2 biosynthetic networks (PV474 and VU192). These 2 networks were chosen as an example to compare similar responsive pathways between the 2 hosts (Fig. 9c). The shared single-copy orthologs between these networks are 3 genes: phenylpropanoid pathway gene C4H (cinnamate-4-hydroxylase), aquaporin protein SIP1, and acyl-activating enzyme 18 (AAE18). The other network genes have connections to shikimate and phenylpropanoid metabolism, but are different specific genes in each species. This shows shikimate and phenylpropanoid metabolism, which are upregulated in both common bean and cowpea, have specific differences in response to Botrytis infection. Thus, even for shared upregulated networks in closely related hosts, the hosts differ in specific network composition.

Some of the upregulated single-copy orthologs in Fig. 8 were from the well-known hormone defense-signaling pathways jasmonic acid (JA) and ethylene. These pathways have previously been shown to be involved in plant defenses against Botrytis. Therefore, we specifically investigated upregulated host GCNs for the presence of JA or ethylene networks. There were 4 single-copy jasmonate-ZIM domain orthologs that were upregulated in both hosts. Of these, none appeared in co-expression networks for common bean. 2 of them, JAZ1 and JAZ6, appeared across 3 GCNs in cowpea. Interestingly, these networks did not include other known and upregulated JA pathway genes (i.e. LOX3, OPR3). For ethylene signaling, there were 3 ERF1 orthologs upregulated in both hosts. Of these, none appeared in co-expression networks for cowpea. One appeared in a network for common bean (PV171), otherwise this network's known members consist of those involved in signaling and transport (Supplementary Table 11). This suggests that these defense response networks are differentiated between common bean and cowpea.

### Most host orthologs respond differently to Botrytis isolates in either host

**Fig. 10.**
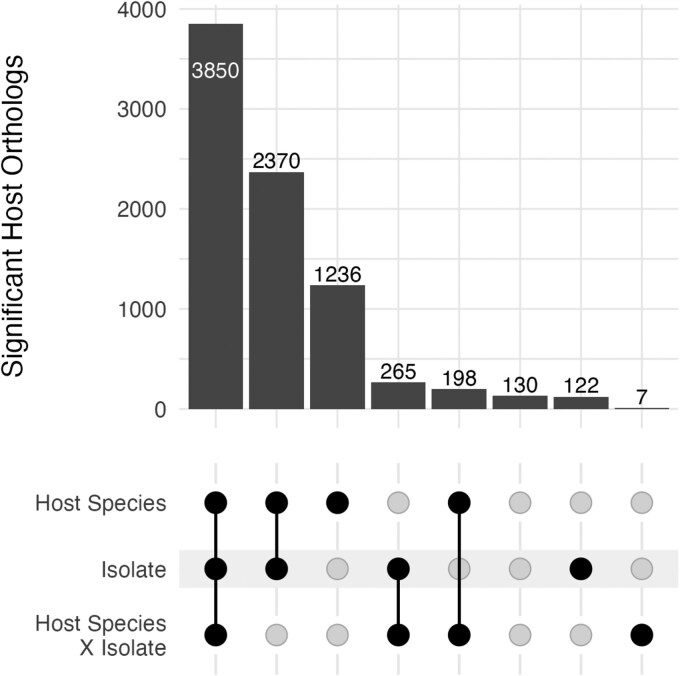
Most host single-copy orthologs show differential expression across hosts and in response to Botrytis. Upset plot summary of ANOVA for each host single-copy ortholog gene expression for legume hosts at 48 HAI. Host orthologs were modeled with the formula: Ortholog expression ∼ Host Species + Isolate + Host Species * Isolate. Genes are included in each category if the FDR-adjusted *P*-value was < 0.05 for that model term. Genes with little to no expression are not diagrammed and include 5,294 host orthologs.

The composition of the host GCNs upregulated in both hosts suggests that while many of the same orthologs are generally upregulated during infection in either host, they co-express differently across hosts, likely depending on both the host and the infecting Botrytis isolate. We tested for these effects by modeling all single-copy orthologs with the following model: Single-copy ortholog expression ∼ host + isolate + host X isolate. This revealed that single-copy genes shared between the hosts largely shift in their expression not only in either host, but also in response to genetic diversity in Botrytis. A large proportion of single-copy orthologs had significant effects across host species, Botrytis isolate, and the interaction (Fig. 10). The vast majority of the expressed orthologs (94%) were differentially expressed between host species, a result expected given that this analysis integrates gene expression from 2 distinct genomes. Despite this strong effect, isolate and host × isolate interaction also contributed substantially to host ortholog expression. Specifically, 81% of the plant orthologs were differentially expressed across isolates, indicating that pathogen genetic diversity broadly shapes host transcriptional responses, and this differs between the 2 hosts. Orthologs that varied across isolates but not the interaction term likely represent responses relatively conserved between hosts. However, over half of host orthologs (53%) had significant host × isolate interactions, indicating isolate-specific effects that differ between hosts. Together, this shows that although these closely related host species share much of their genome, their shared gene expression programs are intricately fine-tuned in response to genetic diversity in a generalist pathogen.

## Discussion

To understand how differences between closely related host species shape interactions with a generalist pathogen, we examined infection outcomes and co-transcriptomic responses of *B. cinerea* and 2 closely related legume hosts, common bean and cowpea. Using a diverse panel of 72 Botrytis isolates, lesion formation and gene expression were quantified in both host and pathogen to separate the effects of host genetic diversity, pathogen diversity, and their interaction (Fig. 1). This identified complex, fine-tuned molecular responses in both host and pathogen that were masked at the phenotypic level.

### Botrytis infection of closely related hosts reveals conserved and divergent molecular interactions

A central question in host generalism is how differences between host species shape molecular interactions during infection. For closely related hosts, this question narrows to (i) whether a generalist pathogen uses similar virulence strategies across hosts, and (ii) whether related hosts respond similarly to the same pathogen. Recent work has shown that Botrytis displays host-specific gene expression across eudicots while maintaining a core of genes expressed across all hosts (Singh et al. 2025). Consistent with this, we observed a mixture of conserved and divergent responses in both host and pathogen transcriptomes, revealing substantial molecular divergence despite close host genetic similarity.

The Botrytis transcriptomic response to closely related hosts was highly host specific, with 90% of the quantifiable transcriptome showing host-dependent expression differences (Fig. 3b). This indicates that Botrytis can sense and differentially respond to subtle differences between hosts. Many of the Botrytis genes differentially expressed between hosts encoded cell wall-modifying enzymes (Fig. 4a), a core component of Botrytis virulence (van Kan 2006). We identified 27 putative Botrytis CAZymes that were differentially expressed during infection of leaves of closely related hosts (Supplementary Table 4). In contrast, prior work using Botrytis isolate B05.10 reported that none of the 1,155 CAZymes encoded in the genome showed host-specific expression on lettuce leaves compared with grape and tomato fruit, showing the utility of using an isolate collection (Blanco-Ulate et al. 2014). Our result indicates cell wall-modifying gene expression during leaf infection may exhibit greater host specificity and responsiveness than previously appreciated. In contrast to these host-specific patterns, expression of some Botrytis specialized metabolic pathways were more conserved between hosts. Biosynthetic gene clusters for the phytotoxins botrydial and botcinic acid showed conserved expression across hosts but varied across isolates (Fig. 5), indicating induction of these major virulence pathways is similar on these 2 hosts. Nevertheless, the full breadth of phytotoxic metabolites produced by diverse isolates of Botrytis remains poorly characterized. We also identified several Botrytis specialized metabolic enzymes differentially expressed between hosts (Fig. 4, a and b), and because small changes in pathway composition can substantially alter final metabolite profiles, it is likely that Botrytis deploys distinct blends of specialized metabolites even when infecting closely related hosts. Together, these results indicate that Botrytis balances conserved virulence strategies with finely tuned, host-specific mechanisms to optimize infection across closely related hosts.

The 2 host species also showed host-dependent differences. Single-copy orthologs between common bean and cowpea enabled direct comparison of the host transcriptional responses to Botrytis. Among expressed host single-copy orthologs, 97% were differentially expressed between the hosts that was largely quantitative (Fig. 10). Despite this extensive divergence, both hosts retained canonical features of the eudicot defense response to necrotrophs. In both hosts, JA and ethylene signaling pathways were upregulated, while photosynthetic machinery was downregulated, consistent with prior studies of Botrytis infection in Arabidopsis (Windram et al. 2012; Zhang et al. 2017, 2019) and lettuce (De Cremer et al. 2013). Phenylpropanoid metabolism was also induced in both hosts, in line with its well-established role in biotic defense (Ranjan et al. 2019; Ninkuu et al. 2025). Alongside these shared pathway-level responses, many single-copy orthologs exhibited opposite regulation between hosts, demonstrating that conserved genes can respond differently to the same pathogen challenge (Fig. 8). These oppositely regulated orthologs spanned diverse functions, with no entire pathways showing consistent opposite regulation. Thus, while major host defense pathways are conserved between closely related hosts, this response is tailored with host-specific gene expression.

### Shared pathways swap gene membership according to host variation

Focusing on co-expression networks in both the host and pathogen, identified GCNs that were differentially modulated between the hosts. These had a similar architecture in which a common core set of genes in the GCN was shared across the 2 hosts, while the extended membership of the module showed extensive gene swapping. This suggests a potential structure for explaining plasticity in host–generalist interactions, whereby instead of full rewiring of pathways depending on the host/attacker, metabolism is shifted around a common core.

Among the Botrytis GCNs, we identified an illustrative example of how gene membership can shift around a common core in a host-dependent manner. Using this example gives an outline of how this particular shift has plausible mechanistic consequences. This Botrytis GCN centers around an NRPS gene cluster on chromosome 12 (Fig. 6). NRPS genes produce nonribosomal peptides, a structurally diverse group of natural products with a broad range of biological activities, including toxins, pigments, and siderophores (Martínez-Núñez and López 2016). NRPS gene clusters are common in fungi, and previous analysis of the B05.10 genome recovered 11 distinct NRPS clusters (Valero-Jiménez et al. 2020). However, little is described about their potential function in Botrytis virulence. Previously, NRPS1 in B05.10 has been shown to confer protection for Botrytis against exogenous toxins, but its deletion actually increased virulence (Fernández-Morales et al. 2021). The Botrytis GCN when infecting common bean contains several key flavonoid precursors and both a PKS and NRPS gene cluster. Other fungi have hybrid PKS-NRPS enzymes that catalyze steps in flavonoid biosynthesis (Zhang et al. 2022). In contrast, on cowpea, this Botrytis GCN only consists of the NRPS gene cluster, and all the shikimate/flavonoid pathway genes are absent. This may indicate that this GCN has a host-dependent modularity in which an NRPS gene cluster can be used for divergent purposes by the same pathogen infecting different hosts.

A similar pattern of core GCNs with modified gene membership was also found when comparing the 2 host species (Fig. 9a). One example was a phenylpropanoid associated GCN that shares a common set of genes across the 2 hosts with a set of distinct genes in each species (Fig. 9c). Although the core enzymatic steps of the phenylpropanoid pathway are conserved across land plants, gene expression is extensively regulated by species-specific transcriptional networks in response to environmental stimuli (Liu et al. 2015). Recent support for the ability of plant regulatory networks to rapidly evolve was evidence that angiosperm gene expression patterns evolve more rapidly than animals (Schuster et al. 2026).

The identification of this pattern in both host and pathogen suggests that shifting regulatory network membership may play a role in the changing relationship between closely related hosts. Complicating this model are some limitations on the utility of network approaches to fully describe biological systems. In particular, it is critical to emphasize that GCNs represent correlations only and do not measure the distance between interacting genes. Additionally, complexities of gene orthology/paralogy complicate mapping GCNs across distant species. The use of closely related species with a high fraction of singly copy orthologs limits but does not obliviate this complication. While the specific mechanistic hypotheses generated in this study will require further validation, these findings show host-dependent transcriptional responses involve network modulation around a common core of genes in both host and pathogen.

### Disease similarity hides molecular variation in both host and pathogen

Frequently, phenotypic surveys are used prior to conducting molecular assays to identify comparisons with the largest changes. This makes an assumption that virulence phenotype and the molecular underpinnings are largely comparable. The Botrytis-bean and Botrytis-cowpea comparison did not support this assumption. While Botrytis lesion formation was similar in these closely related hosts, there were distinct, complex, hidden gene expression differences in both host and pathogen underlying this phenotype. Therefore, fairly dissimilar transcriptomes can lead to similar disease outcomes. The first line of evidence supporting this is that lesion formation was primarily driven by Botrytis isolate variation, with no significant host × isolate interaction (Fig. 2d; Supplementary Table 1), while a majority of both host and pathogen transcripts had a significant host × isolate interaction (Figs. 3b and 10). The second line of evidence is that variation and means for lesion sizes were similar between hosts (Fig. 2a), while host and pathogen transcriptomes showed comparably large divergence between hosts. Botrytis transcriptomes were overall highly distinguishable by the host being infected (Fig. 3a). Finally, the expression of single-copy orthologs between the hosts differed markedly (Fig. 10a).

In the context of host generalism, these results caution against assumptions that similar disease outcomes implicitly arise from conserved molecular interactions. Instead, Botrytis achieves phenotypic consistency through flexible, host-specific transcriptional programs, and likewise hosts execute distinct responses that can converge on similar measures of susceptibility. This pattern is consistent with a convergent evolutionary framework, in which similar disease phenotypes can be maintained by selection on pathogenic success, even when the underlying host–pathogen molecular interactions differ substantially. Under this model, host generalism is enabled not by mechanistic uniformity but by genome-wide transcriptional plasticity in both organisms. We highlight a broader need for co-transcriptome studies to strengthen this model and continue enhancing our understanding of the biology underlying generalist pathogens.

## Materials and methods

### Plant materials and growth conditions

To test how genetic diversity within closely related hosts of Botrytis influences disease outcome, 4 genotypes for each of 2 related host species, common bean (*P. vulgaris*) and cowpea (*V. unguiculata*) were included. Genotypes were sourced from the Gepts lab at UC Davis and included UC Cran, UC Tiger's Eye, Black Nightfall, and Orca for common bean, and CB27, CB46, Sanzi, and IT97K-499-35 for cowpea. All plants in this study were grown in the same greenhouse with 16 h of supplemental lighting, 30% to 80% humidity, and a temperature of 65 to 80 °F. Seeds were germinated and grown in Agronomy Mix, and drip irrigated 5 times a day. For lesion phenotyping, plants were grown for 2 independent trials, one in May 2022 and one in July 2022. For transcriptomics, common bean was grown in January 2024, while cowpea was grown in April 2024. Plants were grown for the formation of several true leaves, and before flowering, approximately 4 weeks.

### *B. cinerea* materials and growth conditions

A set of 72 Botrytis isolates was used in this study, as described previously (Singh et al. 2025). This set of 72 samples the genetic diversity and virulence of a larger population of 96 Botrytis isolates, originally isolated as single spores from plant tissues collected mainly from California and internationally (Zhang et al. 2017, 2019; Caseys et al. 2021). There is no evidence of population structure by host or geography in this collection of isolates (Atwell et al 2018). Isolates were stored at −80 °C as a spore stock in 60% glycerol. For experiments, spores were diluted 1:10 (v/v) glycerol stock in sterile grape juice and grown on potato dextrose agar for 2 weeks to obtain spores for infection assays.

### Detached leaf assay

To measure the virulence of Botrytis and hosts within the legumes, the above 8 host genotypes of common bean and cowpea were infected with 72 Botrytis isolates using a previously described detached leaf assay (Denby et al. 2004; Corwin et al. 2016; Soltis et al. 2019). This used a randomized complete block design with 3 independent replicates for each host–genotype combination. Performed across 2 independent experiments, this yielded 6 replicates of lesion phenotype data for each host–pathogen genotype combination.

For each above independent experiment, leaves were collected from approximately 40 plants for each genotype and randomly distributed across trays containing 1 cm of 1% phytoagar with humidity domes at room temperature. Inoculum was generated by growing spores on PDA plates for 10 to 14 d, collecting spores into water, then diluting them into 50% grape juice to 10 spores/μL. Grape juice was used as the inoculum medium to promote consistent germination across isolates. This alleviates issues where Botrytis has genetic variation impacting germination on single sugar sources (Blakeman 1975; Clark 1977; Benito et al. 1998; Denby et al. 2004). Detached leaves were inoculated on 2 sites on either side of the adaxial leaf surface (Fig. 1) with 2 different Botrytis isolates.

### Lesion measurement and analysis

To measure the developing lesions, images of trays containing inoculated leaves were collected at 24, 48, 72, and 96 HAI. At 72 HAI, lesion formation was visible and quantifiable on leaves. For 72 and 96 HAI images, lesion areas were traced digitally using the EBImage and CRImage packages (Pau et al. 2010; Failmezger et al. 2012) in a previously described R pipeline (Fordyce et al. 2018), then manually inspected to verify accuracy. Lesion areas in pixels were converted to mm^2^ using a reference scale included within each image.

Lesion area was modeled using the lme4 package (Bates et al. 2015). We modeled lesion area according to the host, pathogen, and their interactions while accounting for experimental design with the following:

Lesion Size ∼ Species + Species/Genotype + Isolate + Species * Isolate + Species/Genotype * Isolate + (1|Tray) + (1|Trial)

Host genetic diversity, including both “Species” and “Species/Genotype” (genotype nested within species), pathogen genetic diversity as “Isolate,” and their interactions were treated as fixed effects. Experimental design components, including “Tray” (phytoagar flat containing inoculated leaves within a randomized block design) and “Trial” (independent experiment), were treated as random effects.

### RNAseq library preparation and sequencing

To measure the associated gene expression patterns of both host and pathogen during infection, the above detached leaf assay was conducted with 1 selected genotype each from common bean and cowpea. The host genotypes were selected based on a number of factors, including range of infection phenotypes across the Botrytis isolates, available reference genomes, and ease of healthy plant growth. The detached leaf assay was performed as 1 independent experiment for each host species, yielding 3 replicates of transcriptomic data.

At 48 HAI, a 1.20 cm diameter leaf disc containing the inoculation site was removed and immediately flash frozen in liquid nitrogen. Mock leaf samples that were inoculated with grape juice only were also collected at 48 HAI. The 48 HAI timepoint was selected based on a pilot experiment conducted at 24, 30, 36, and 48 HAI, which showed that 48 HAI yielded the highest proportion of fungal-mapped reads. Inoculated leaf discs were stored at −80 °C until processing. Libraries were prepared according to a previously described method (Kumar et al. 2012; Zhang et al. 2017). RNA extraction was conducted by placing samples in liquid nitrogen and then homogenizing by rapid agitation in a bead beater, followed by direct mRNA isolation using the Dynabeads oligo-dT kit. First- and second-strand cDNA was produced from the mRNA using an Invitrogen Superscript III kit. The resulting cDNA was fragmented, end-repaired, A-tailed, and barcoded as previously described. Adapter-ligated fragments were enriched by PCR and size-selected using AMPure XP beads for a mean of 300 bp prior to sequencing. Barcoded libraries were pooled in batches of 74 (1 host species inoculated with 72 isolates and 2 mocks) and submitted for paired-end 150 bp sequencing on a single lane per pool using the AVITI sequencing platform at the UC Davis Genome Center (DNA Technologies Core, Davis, CA).

### Transcriptomic data analysis

The resulting fastq files were separated by adapter index into individual sample files. Quality control was done with MultiQC (Ewels et al. 2016). Raw paired-end sequencing reads were quality-trimmed and filtered using Trimmomatic (Bolger et al. 2014). Adapter sequences were clipped using the ILLUMINACLIP module, allowing up to 2 seed mismatches, a palindrome clip threshold of 30, and a simple clip threshold of 10. Reads were further processed to remove low-quality bases from the 5′ and 3′ ends, trim within a sliding window of 4 bases where the average quality dropped below 15, and discard reads shorter than 20 bases. Trimmed and paired reads were retained for downstream analysis, and quality was reassessed with MultiQC. Reads were mapped to reference genomes using Hisat2 (Kim et al. 2019). Bulk reads were mapped first to the host genome (Lonardi et al. 2019; Schmutz et al. 2014). The remaining unmapped reads were mapped to Botrytis B05.10 isolate reference genome (Van Kan et al. 2017). Read counts were obtained from the mapped reads using RSubread (Shi 2017). Read counts were TMM normalized with the EdgeR package (Robinson et al. 2010). Low-expression genes (genes with <1 CPM in <20% of samples within each host) were removed from the dataset. All statistical analyses were conducted within R.

To estimate the effects of host and pathogen on all transcripts, a generalized linear model with a negative binomial distribution (log link) was fitted using the glmmTMB R package. Host gene expression was modeled for each host gene across both species with the following:

Host gene expression ∼ Infected + Infected/Botrytis Isolate + (1|Tray) + (1|Sequencing Batch)

Infection status, including both “Infected” (inoculated with any Botrytis vs mock) and “Infected/Botrytis Isolate” (Botrytis isolate nested within infected), was treated as fixed effects. Experimental design components, including “Tray” (phytoagar flat containing inoculated leaves within a randomized block design) and “Sequencing Batch” (sample set prepared and sequenced together), were treated as random effects.

For the Botrytis transcripts, both hosts were included in the same model. The effects of host species, Botrytis isolate, and their interaction were modeled using the following formula:

Botrytis gene expression ∼ Host Species + Botrytis Isolate + (Host Species * Botrytis isolate) + (1|tray) + (1|sequencing batch)

Estimated marginal means and standard error for each transcript were determined for each host genotype & Botrytis isolate using the emmeans package (Lenth and Piaskowski 2017). Model results were summarized using type II Wald chi-square tests from the car package to evaluate the significance of each fixed effect. Resulting *P*-values were adjusted for multiple comparisons using the false discovery rate (FDR) correction (Benjamini and Yekutieli 2001).

PCA was conducted in R's base package (R Core Team 2024). To evaluate the correlation of Botrytis lesion size and Botrytis transcriptome abundance to principal components, the following model was used:

Botrytis lesion size ∼ PC1 + PC2

To assess enrichment of certain gene classes in differentially regulated Botrytis genes, hypergeometric tests were performed with the stats package in R. *P* values were calculated for both overenrichment and underenrichment of the gene class for a given regulation group, then FDR adjusted.

### Co-expression network analysis

To assess gene co-expression patterns both within and across host and pathogen, we used a previously described gene co-expression network analysis pipeline (Wisecaver et al. 2017). For each host–pathogen combination, normalized gene counts from expressed genes were estimated separately from host and pathogen transcripts. Normalized host and pathogen transcriptomes were then combined and used to calculate correlations among all transcripts. The pipeline was run twice: once for common bean infected with Botrytis and once for cowpea infected with Botrytis. Pearson correlation coefficient values for each gene pair were calculated, then scored for mutual rank. Mutual ranks were used to call modules of tightly co-expressed genes using ClusterONE with a decay rate of 5 (Nepusz et al. 2012). Networks with at least 10 gene members and a significant network correlation statistic (*P* < 0.05) were retained for further analysis. This yielded 378 total host & Botrytis networks on common bean, and 428 total networks of host & Botrytis networks on cowpea. Across this set of networks, the average number of genes per network was 18. For gene co-expression networks of interest, overall network expression level was calculated by z-scaling expression values within each gene in the dataset and taking the mean expression across genes in the network.

### Host legume orthology analysis

To compare responses to Botrytis directly between the 2 host species, orthology analysis was conducted with Orthofinder (Emms and Kelly 2019). Briefly, this analysis used predicted proteomes from each host species to identify orthogroups across the 2 species. The algorithm then infers gene trees, a rooted species tree, and then analyzes the rooted gene trees to identify orthologs and gene duplication events. This results in a full list of predicted orthologs between the species, including single-copy orthologs and any duplicated genes.

To estimate the main functions of host single-copy orthologs from Orthofinder grouped by their response to infection vs mock, gene ontology (GO) enrichment analysis was performed using the R package topGO (Adrian Alexa 2017). This involved 4 separate tests: GO enrichment of genes upregulated in both hosts, genes downregulated in both hosts, genes upregulated in common bean but downregulated in cowpea, and genes upregulated in cowpea but downregulated in common bean. The analysis tested for enrichment of terms from the Biological Process ontology among differentially expressed single-copy orthologs, using all single-copy orthologs as the background. Enrichment was assessed with Fisher's exact test under the classic algorithm, and terms were ranked by *P*-values.

To assess the relative contribution of host and Botrytis genetic diversity to the expression of host single-copy orthologs, a generalized linear model with a negative binomial distribution (log link) was fitted using the glmmTMB R package:

Ortholog expression ∼ host + isolate + host × isolate

Model significance was evaluated using type II Wald chi-square tests implemented in the car package. Resulting *P*-values were adjusted for multiple comparisons using the FDR correction (Benjamini and Yekutieli 2001). Variance components were calculated from sums of squares.

## Supplementary Material

jkag125_Supplementary_Data

## Data Availability

All data supporting this study are included in the main article and/or the supplementary information. The RNA-seq data are available in the NCBI Gene Expression Omnibus (GEO) under accession GSE329643 and in the NCBI Sequence Read Archive (SRA) under BioProject ID PRJNA1217477. Supplemental material available at *G3* online.
