# Supplementary material for: Host–Botrytis co-transcriptomics reveals finely tuned interactions with closely related legumes
Source: G3 (Bethesda). 2026 May 12;16(7):jkag125. doi: 10.1093/g3journal/jkag125 (PMC13334180; doi:10.1093/g3journal/jkag125)
Supplement: jkag125_Supplementary_Data [file jkag125_supplementary_data.zip › Supplemental_Material_G3-2026-406670.docx]

# **Supplemental Material**

**Table S1** ANOVA of lesion sizes during *Botrytis cinerea* infection of *Phaseolus vulgaris* and *Vigna unguiculata*.

**Table S2** Sequence and mapping information for all RNAseq samples collected in the study.

**Table S3** GO enrichment of the 58 solely host effect genes against the whole *Botrytis cinerea* genome when infecting *Phaseolus vulgaris* and *Vigna unguiculata*.

**Table S4** Differential expression analysis of all *Botrytis cinerea* genes across 2 host legume species (*Phaseolus vulgaris* and *Vigna unguiculata*).

**Table S5** Significantly enriched GO terms of the 422 Botrytis genes upregulated during infection of common bean and the 656 Botrytis genes upregulated during infection of cowpea.

**Table S6** 18 identified *Botrytis cinerea* GCNs with isolate specificity, but similarly expressed across hosts (*Phaseolus vulgaris* and *Vigna unguiculata*).

**Table S7** Gene lists for the main phytotoxic gene clusters in *Botrytis cinerea*, botrydial and botcinic acid.

**Table S8** Lesion sizes and mean expression level of key *Botrytis cinerea* phytotoxin clusters for each combination of *B. cinerea* isolate and host species (*Phaseolus vulgaris* and *Vigna unguiculata*).

**Table S9** Differential expression analysis showing Mock to Infected log2FC of all host genes in the study.

**Table S10** GO enrichment of legume host single copy orthologs in *Phaseolus vulgaris* and *Vigna unguiculata* when infected with *Botrytis cinerea*.

**Table S11** 37 *Botrytis cinerea* infection-responsive host GCNs containing >=50% single copy orthologs between common bean (*Phaseolus vulgaris*) and cowpea (*Vigna unguiculata*).

**Figure S1.** **Least-squared means of lesion sizes of 72 Botrytis isolates on legume hosts over time.** Each isolate is shown as a different colored line and its lesion value is averaged across 8 host genotypes. HAI = Hours after inoculation.

Alt text: Plot showing change in lesion size (mm^2^) for each isolate from 72 HAI to 96 HAI. Each isolate is shown as a different colored line.

**Figure S2. Comparative lesion size residuals of specific isolates as a metric for specialism of each isolate.** Genotype residuals were calculated as the mean of one isolate on a given genotype subtracted from the overall mean of that isolate across the species. Species-level residuals were then calculated for each isolate by taking the absolute value sum of the four genotype residuals for each species. Outlying isolates are labeled with the isolate name. Correlation statistics: R^2^ = 0.458, p < 0.01.

Alt text: Scatter plot showing Botrytis isolates lesion residuals (mm^2^) and trendline when infecting common bean (on the x axis) and cowpea (on the y axis).

**Figure S3. PCA of overall Botrytis transcriptome at 48 HAI.** Shape of points show the species the infecting Botrytis isolate was collected from, where circles are common bean and triangles are cowpea. Points are colored by total transcript abundance of that isolate on that host species at 48 HAI.

Alt text: A scatter plot showing principal component analysis (PC1 vs. PC2) of Botrytis transcriptomes, with points colored by Botrytis transcript abundance and different shapes for host species (circles as common bean and triangles as cowpea).

**Figure S4. Individual Botrytis gene and network expression do not strongly contribute to lesion size.** a) Linear model results assessing the relationship between individual Botrytis gene expression and lesion size each gene’s correlation to lesion size (Lesion Size ~ Host + Gene Expression + Host * Gene Expression). Proportion of variance explained (R^2^) and significance (-log_10_ p value) are shown for each gene’s expression (left) and host x expression interaction (right). The dotted line denotes the significance threshold (p < 0.05). b) Mean expression of Botrytis gene networks in isolates associated with small versus large lesions. Network expression was calculated as the mean expression of all genes within each network for a given isolate. The top 10 and bottom 10 isolates ranked by lesion size (large and small lesion groups, respectively) were selected, and mean network expression was calculated for each network within each group. The NRPS networks discussed in Figure 6 are labeled.

Alt text: Four scatter plots where a) shows R^2^ on the x axis and -log_10_ p value on the y axis for the gene’s expression (left) and host x expression interaction (right). The lower two scatter plots show mean network expression values of small lesion isolates on the x axis and large lesion isolates on the y axis, with b) infecting common bean and c) infecting cowpea.

**Figure S5. Selected Botrytis co-expression networks containing several Botrytis genes with significant host x isolate interaction.** Overlapping Botrytis gene co-expression networks were detected when infecting both a) common bean and b) cowpea with slight differences. The network represents co-expression of a Botrytis gene cluster on chromosome 13. C) Z-scaled expression of these interaction networks on different hosts. Colored points and connecting lines represent different Botrytis isolates.

Alt text: Two Botrytis gene co-expression networks when infecting common bean (a) and cowpea (b). A third plot shows z-scaled network expression of each network on each host.

**Figure S6. PCA of host transcriptomes colored by a) Botrytis transcript abundance and b) lesion size at 96 HAI in mm^2^.** Each point represents a Botrytis isolate on either common bean or cowpea. Isolate colored grey represents the mock inoculation. PC1 accounts for 42% of the variance in cowpea, 39% variance in common bean; PC2 accounts for 8% of the variance in cowpea, 7% variance in common bean.

Alt text: Two sets of scatter plots (a and b) showing principal component analysis (PC1 vs. PC2) of samples for common bean and cowpea, with points colored by Botrytis transcript abundance in (a) and lesion size (mm²) in (b).

**Figure S7.**  **Expressed gene ortholog summary between common bean and cowpea.** Single copy (1:1) orthologs are those that have a single match in either legume species, while 1:many orthologs are those that have one gene in one species that match >1 in the other species, etc. Gene counts are colored by their significance in the model gene expression ~ infected + infected/isolate. “Infected only” are genes only significant in the *infected* main effect. “Isolate only” are genes only significant in the *infected/isolate* (nested) term. “Infected and isolate” are genes that are significant for both the *infected* main effect and the *infected/isolate* term.

Alt text: Two stacked bar plots showing the number of genes by orthology category for common bean (left) and cowpea (right), with bars divided into significance groups (infected and isolate, infected only, isolate only, and no effect) and the y-axis representing gene counts.

**Figure S8. Differential expression of host genes in response to infection with Botrytis cinerea separated by orthology group.** Highlighted genes have a significant response to infection (p < 0.05) and mock to infected log2FC >= 1 or <= -1.

Alt text: Four volcano scatter plots showing differential gene expression with log2 fold change (mock vs. infected) on the x-axis and –log10(p-value) on the y-axis for common bean (top) and cowpea (bottom), separated into genes with no orthology (left) and single-copy orthologs (right), with points colored by significance (red: upregulated, blue: downregulated, gray: not significant).
