# Supplementary figures and images for: Host–Botrytis co-transcriptomics reveals finely tuned interactions with closely related legumes
Source: G3 (Bethesda). 2026 May 12;16(7):jkag125. doi: 10.1093/g3journal/jkag125 (PMC13334180; doi:10.1093/g3journal/jkag125)

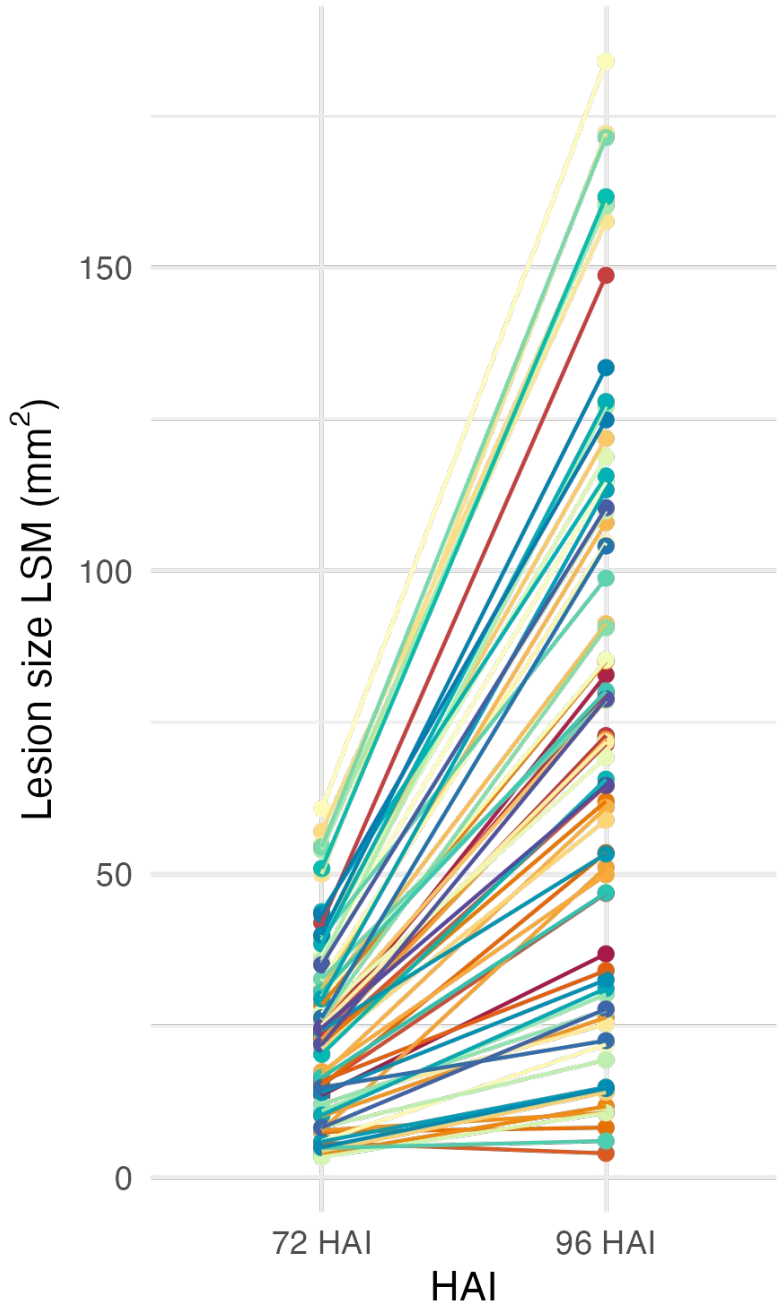

Supplement: jkag125_Supplementary_Data [file jkag125_supplementary_data.zip › Figure_S1_G3-2026-406670.pdf]

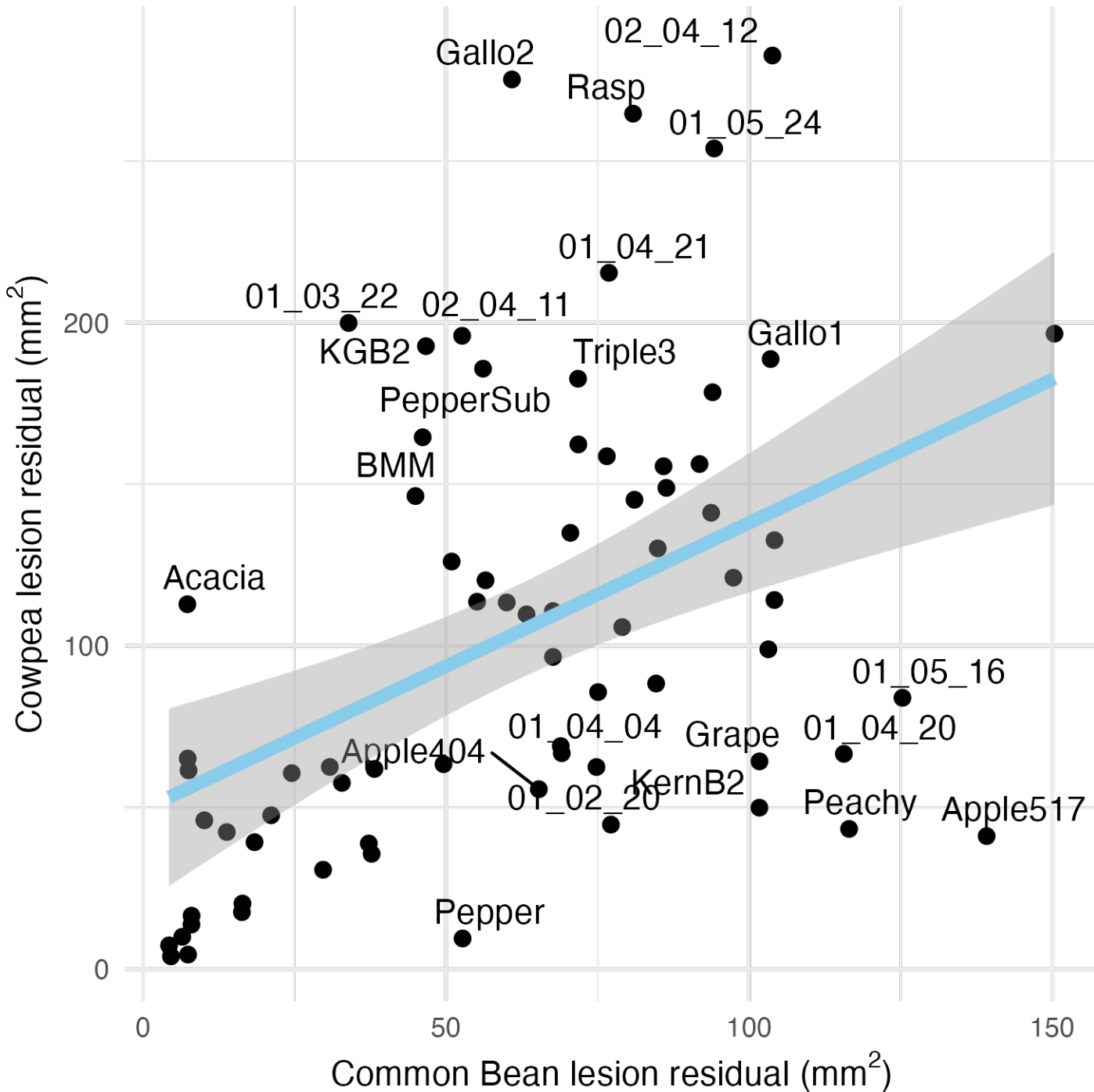

Supplement: jkag125_Supplementary_Data [file jkag125_supplementary_data.zip › Figure_S2_G3-2026-406670.pdf]

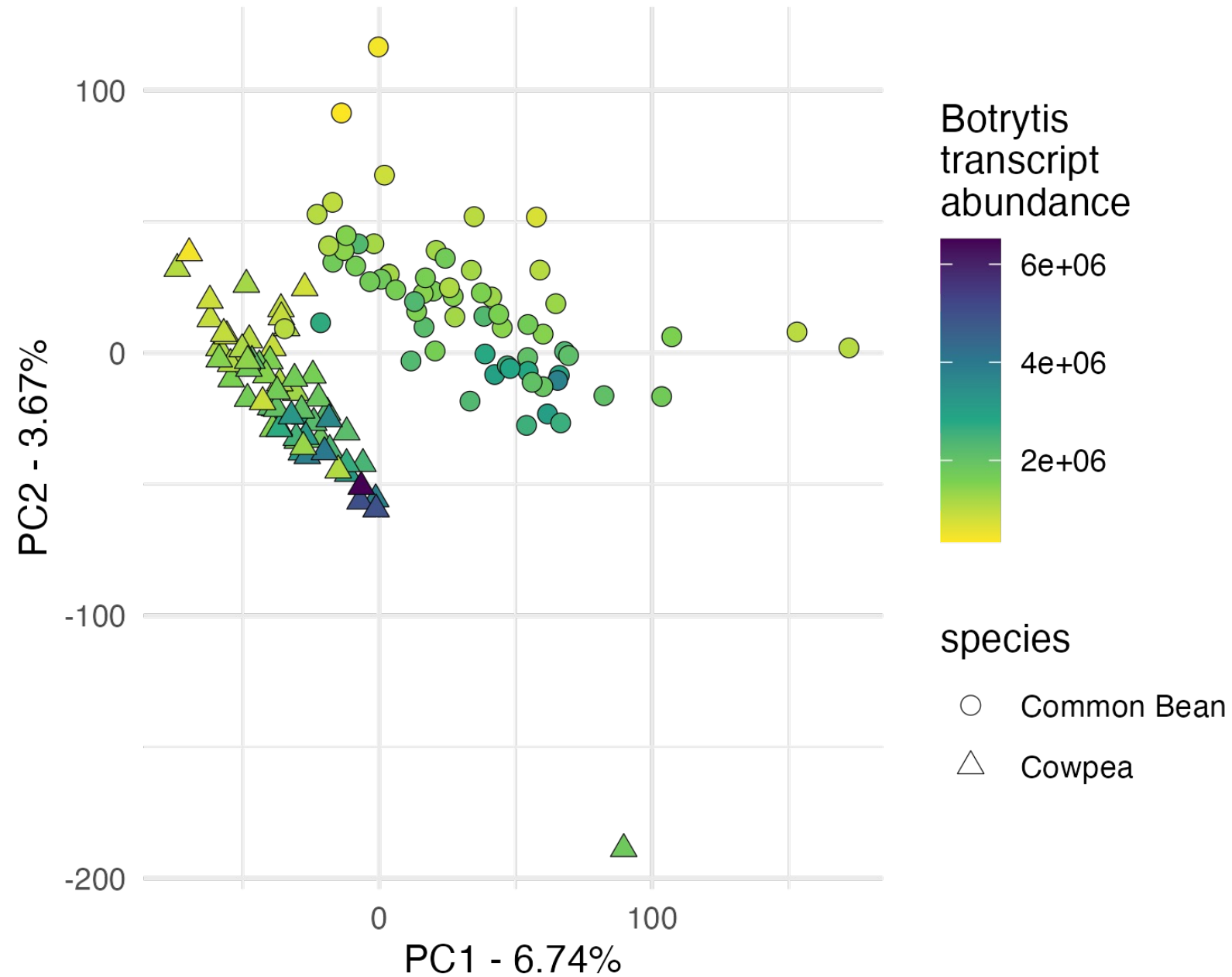

Supplement: jkag125_Supplementary_Data [file jkag125_supplementary_data.zip › Figure_S3_G3-2026-406670.pdf]

**a**

Gene Expression

Gene Expression x Host

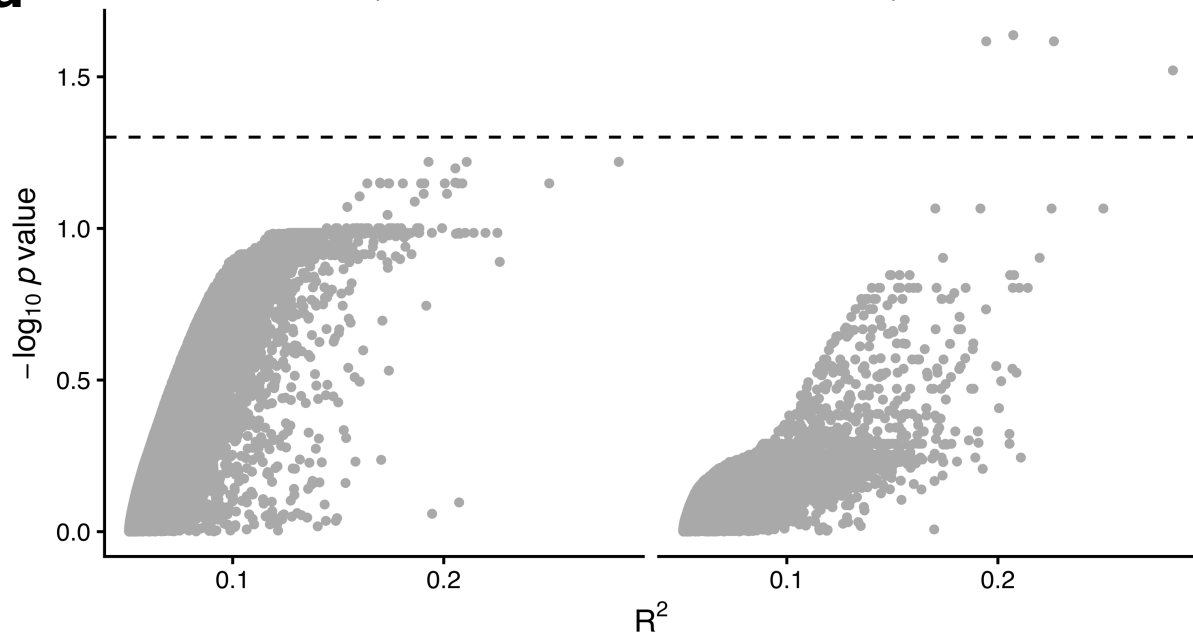**b**

Infecting common bean

Infecting cowpea

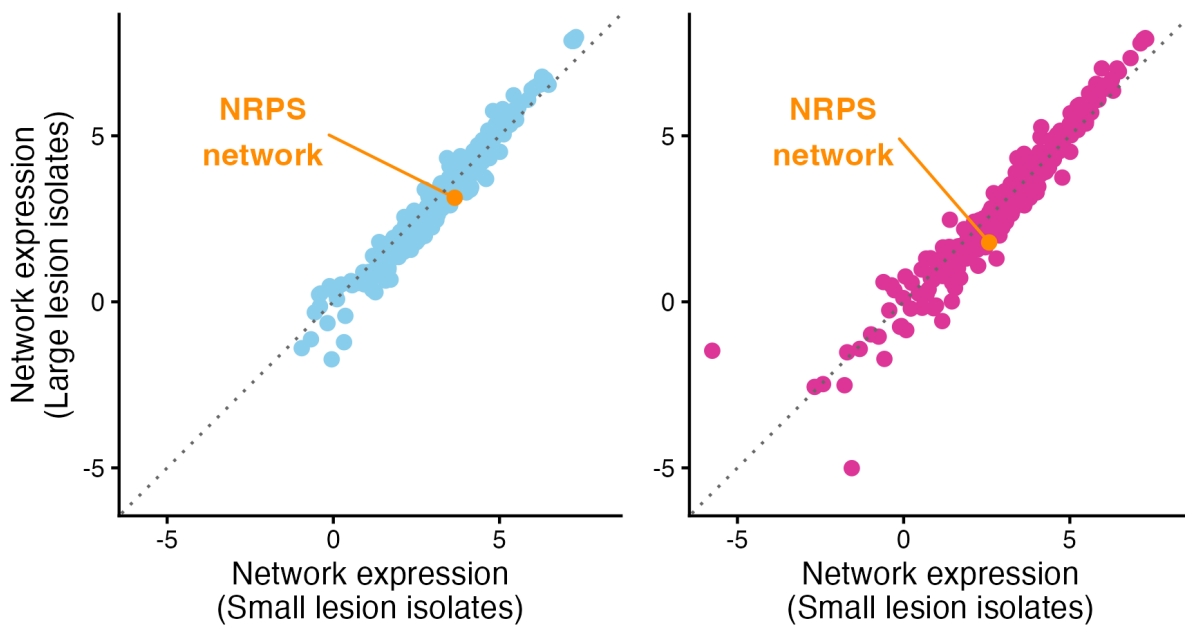

Supplement: jkag125_Supplementary_Data [file jkag125_supplementary_data.zip › Figure_S4_G3-2026-406670.pdf]

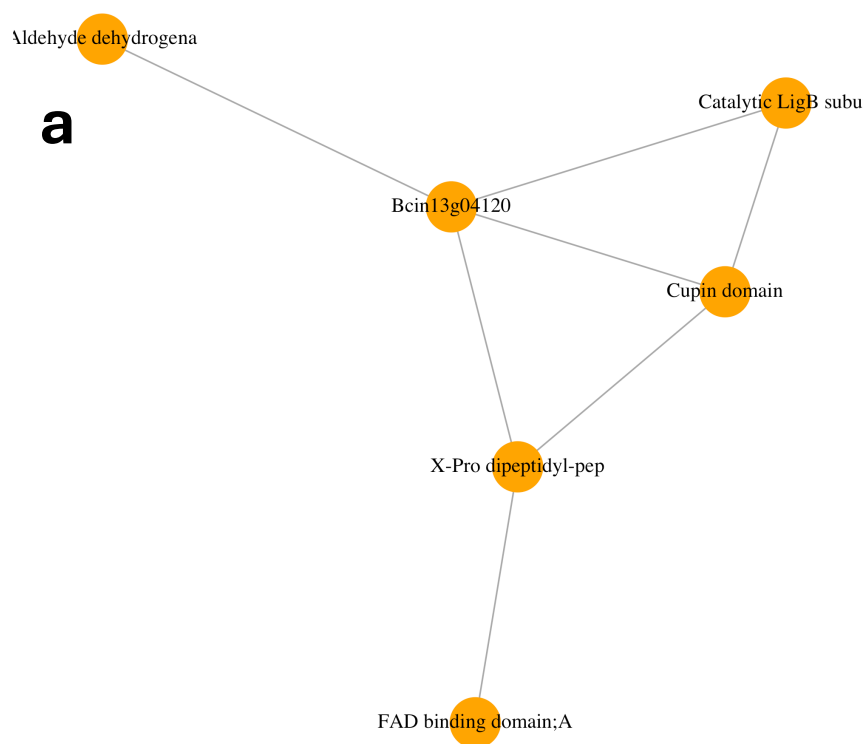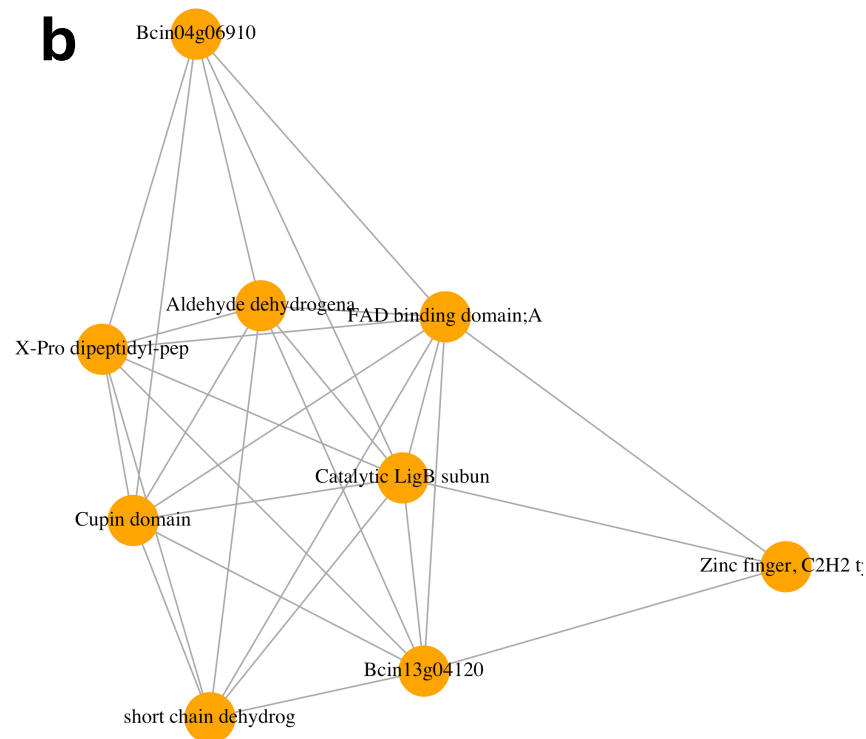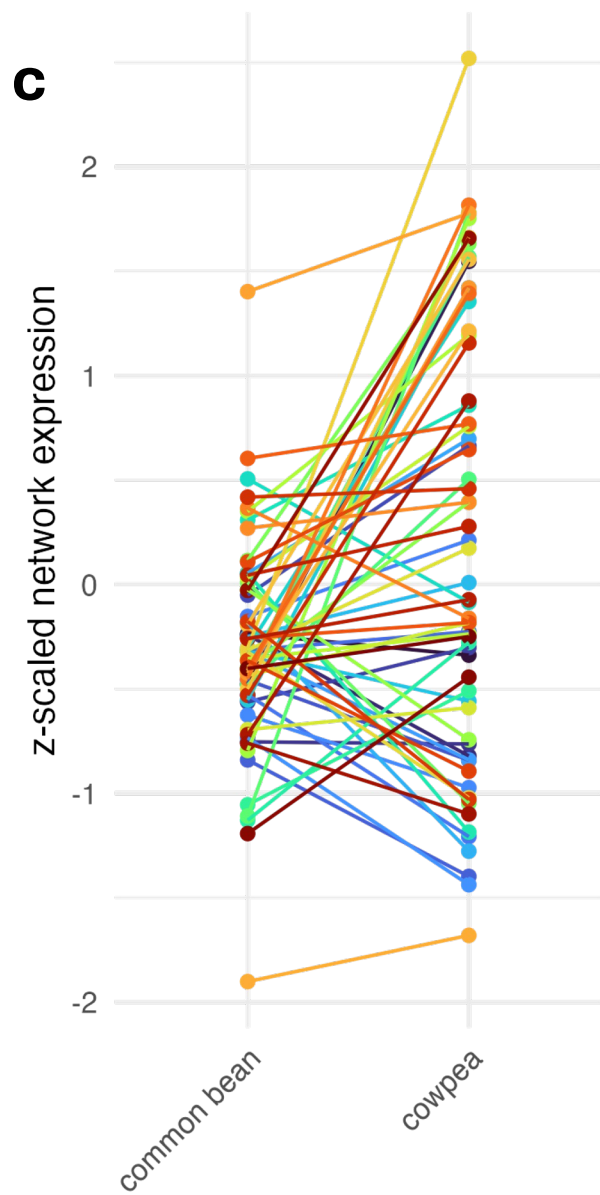

Supplement: jkag125_Supplementary_Data [file jkag125_supplementary_data.zip › Figure_S5_G3-2026-406670.pdf]

**a**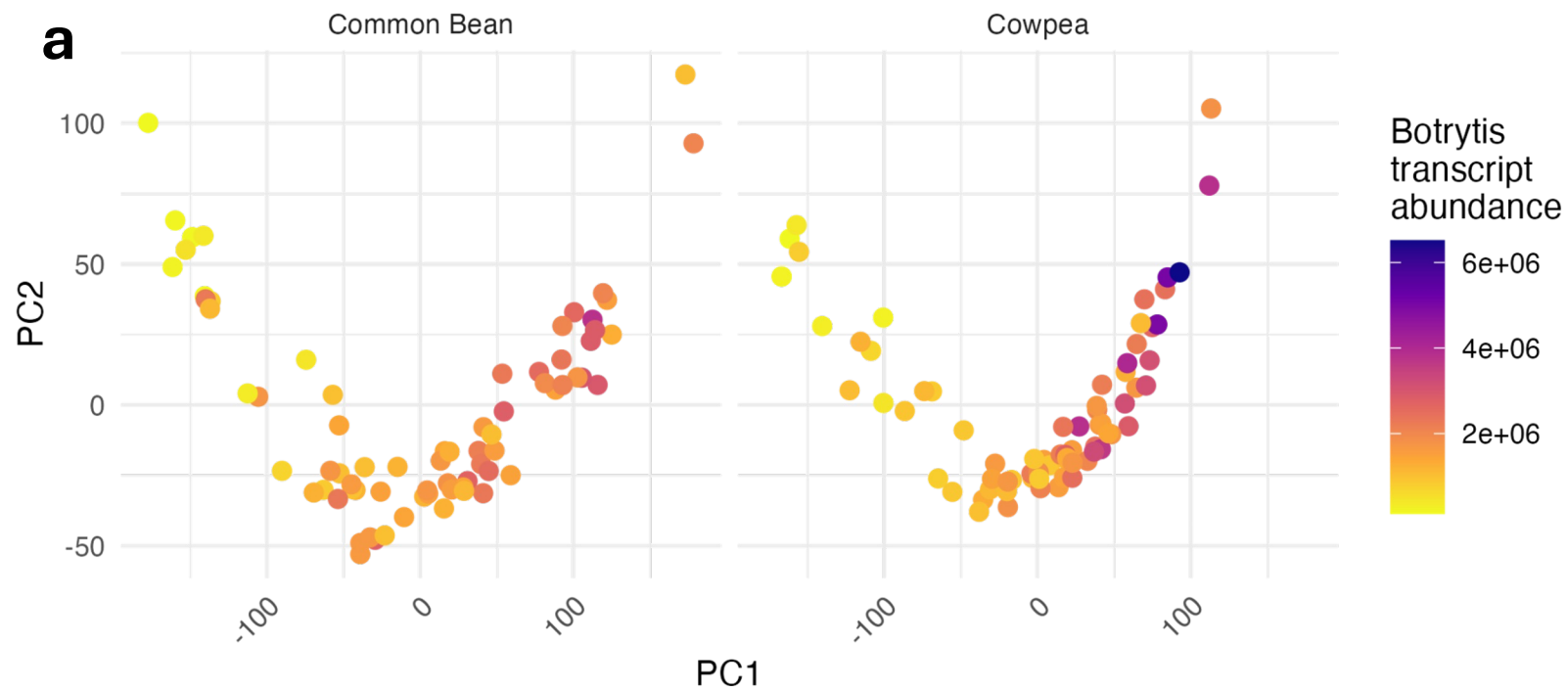**b**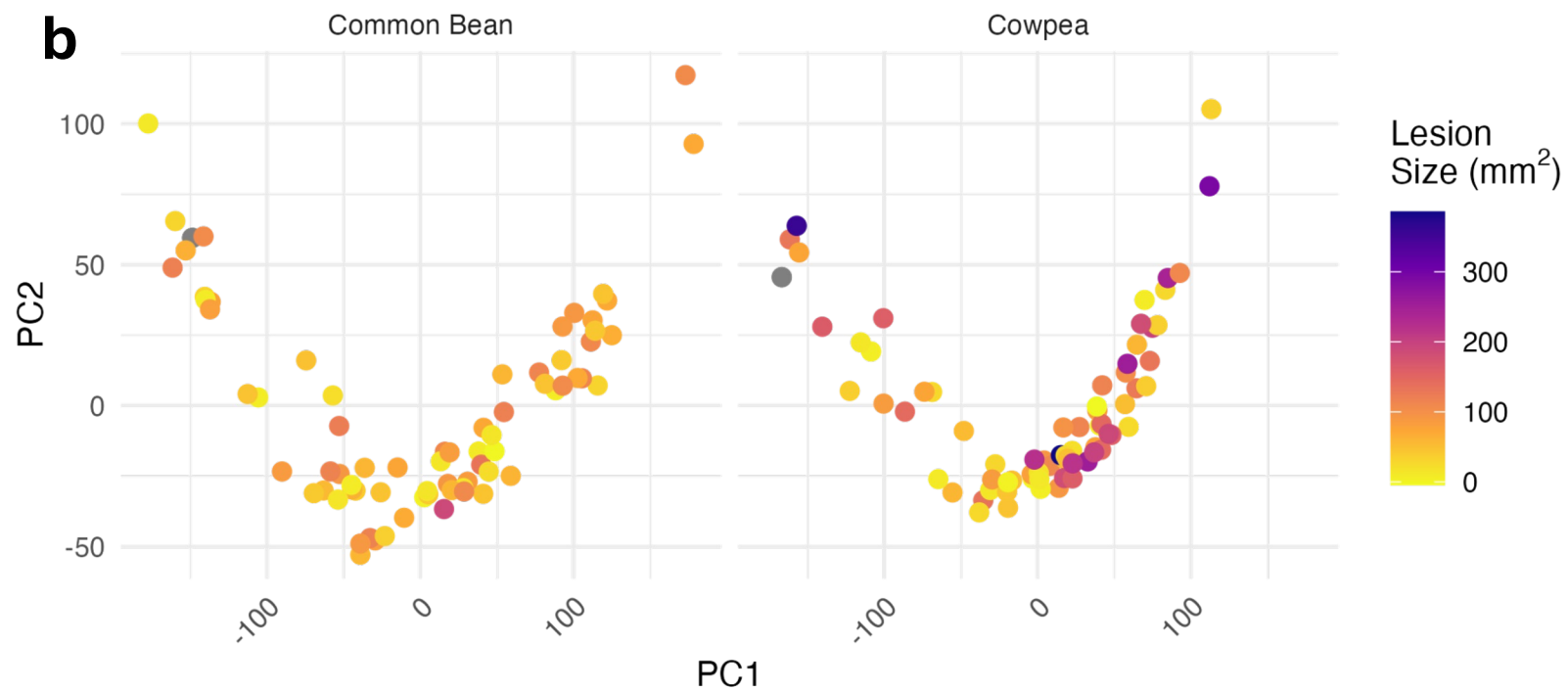

Supplement: jkag125_Supplementary_Data [file jkag125_supplementary_data.zip › Figure_S6_G3-2026-406670.pdf]

## Common Bean

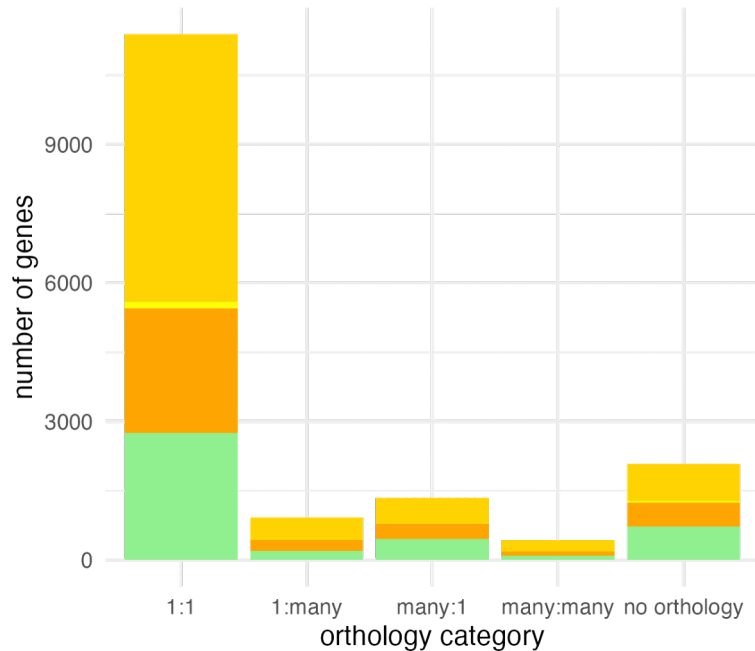

## Cowpea

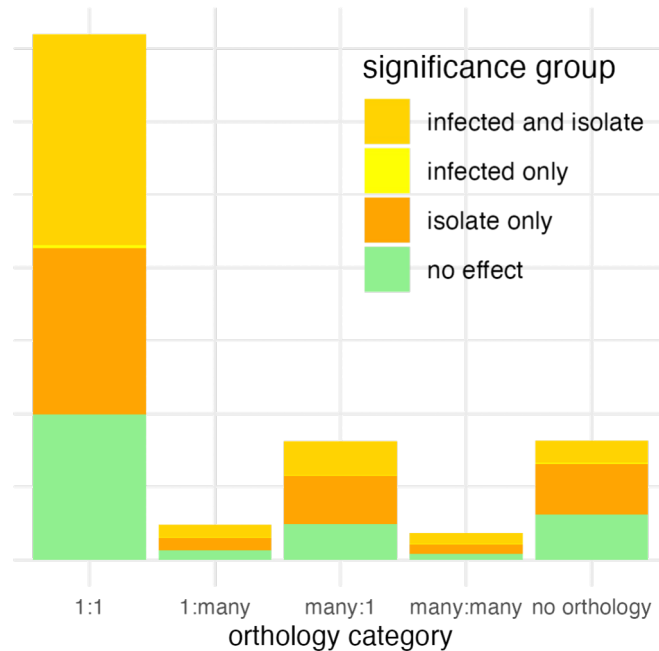

Supplement: jkag125_Supplementary_Data [file jkag125_supplementary_data.zip › Figure_S7_G3-2026-406670.pdf]

Common bean

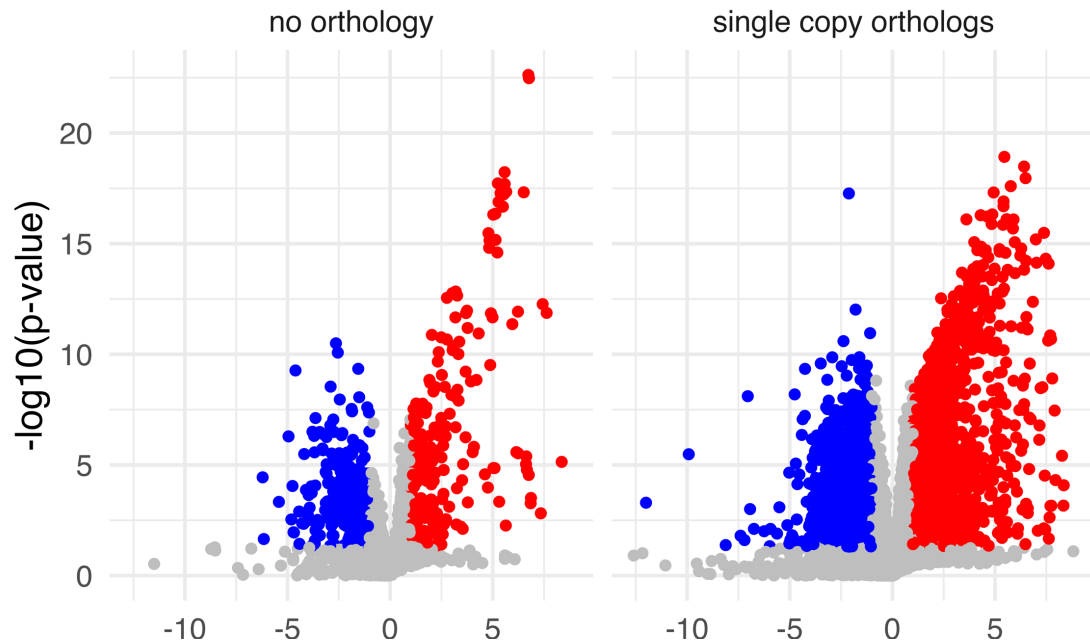

Cowpea

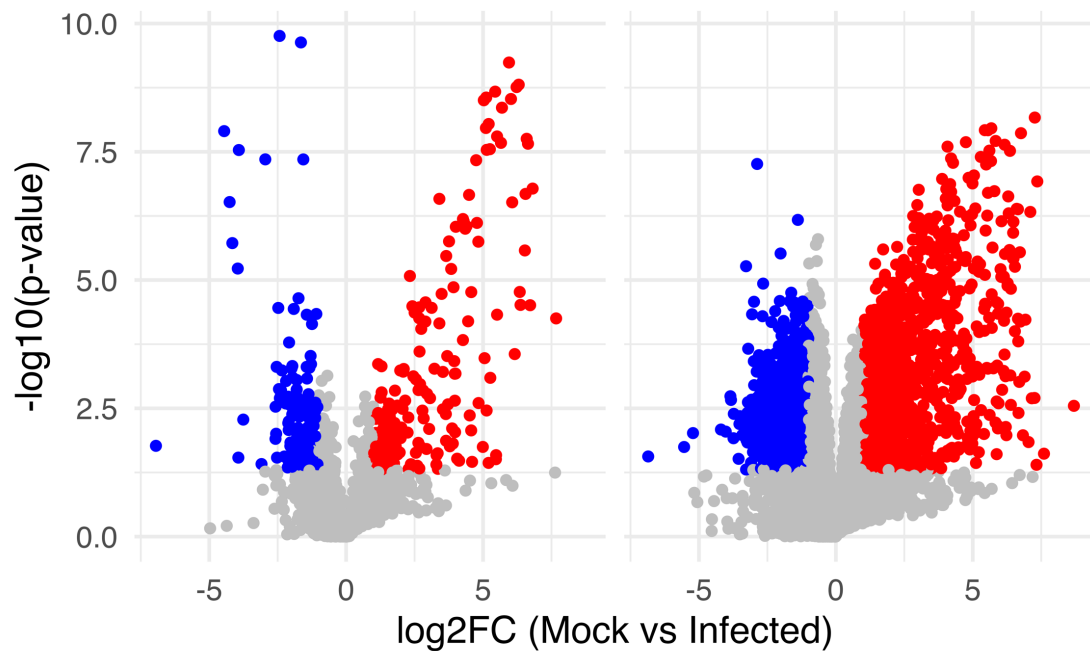

Supplement: jkag125_Supplementary_Data [file jkag125_supplementary_data.zip › Figure_S8_G3-2026-406670.pdf]
